# Synthesis and PI 3-Kinase Inhibition Activity of Some Novel 2,4,6-Trisubstituted 1,3,5-Triazines

**DOI:** 10.3390/molecules23071628

**Published:** 2018-07-04

**Authors:** Ronald A. Nelson, Taylor Schronce, Yue Huang, Alanoud Albugami, George Kulik, Mark E. Welker

**Affiliations:** 1 Department of Chemistry, Wake Forest University, Winston-Salem, NC 27109, USA; ranelson@wakehealth.edu (R.A.N.J.); taylorschronce@alumni.wfu.edu (T.S.); 2 Department of Cancer Biology and Comprehensive Cancer Center, Wake Forest School of Medicine, Medical Center Blvd., Winston-Salem, NC 27157, USA; yhuang@wakehealth.edu; 3 Life Sciences program, College of Science, Alfaisal University, Riyadh 11533, Saudi Arabia; aalbugumi@alfaisal.edu

**Keywords:** triazine synthesis, PI3K inhibitor, prostate cancer

## Abstract

A number of new trisubstituted triazine phosphatidylinositol 3-kinase (PI3K) inhibitors were prepared via a three-step procedure utilizing sequential nucleophilic aromatic substitution and cross-coupling reactions. All were screened as PI3K inhibitors relative to the well-characterized PI3K inhibitor, ZSTK474. The most active inhibitors prepared here were 2–4 times more potent than ZSTK474. A leucine linker was attached to the most active inhibitor since it would remain on any peptide-containing prodrug after cleavage by a prostate-specific antigen, and it did not prevent inhibition of protein kinase B (Akt) phosphorylation, and hence, the inhibition of PI3K by the modified inhibitor.

## 1. Introduction

Androgen ablation therapy remains the most successful systemic treatment for prostate cancer. In most cases, the disease initially responds, but it eventually recurs as advanced androgen-independent prostate cancer, for which no effective treatment is currently available. Prostate cancer cells that acquire androgen independence become resistant to chemotherapy and radiation therapy [1,2,3]. To further extend the survival of prostate cancer patients, it is necessary to understand the mechanisms that underlie the responses of prostate cancer cells to therapies. 

Normal prostate epithelial cells undergo apoptosis after androgen levels decrease [4]. It was proposed that, in order to survive, androgen-independent prostate cancers activate androgen receptors (ARs) by very low levels of androgen, or transactivate ARs in the absence of androgen [5,6]. Several therapeutic strategies for complete androgen ablation were developed, including combinations of gonadotropin-releasing hormone analogs (e.g., leuprolide) that cause continuous stimulation of the pituitary gland, abiraterone acetate (an inhibitor of androgen biosynthesis), and enzalutamide (MDV3100), an AR antagonist that prevents the binding of androgens to AR, nuclear translocation, and chromatin binding of AR. However, even with complete blockage of AR signaling, disease eventually progresses [7,8]. Accumulating evidence suggests that advanced prostate cancer engages androgen-independent signal transduction pathways that inhibit apoptosis, and hence, “bypass” the requirement for AR activation [9,10].

Numerous publications emphasize the role of anti-apoptotic signaling via protein kinase cascades in the therapeutic resistance of advanced androgen-independent prostate cancer, among which the phosphatidylinositol 3-kinase/protein kinase B (PI3K/Akt) pathway gained prominence [10,11,12,13,14]. Continuous activation of PI3K/Akt signaling allows prostate tumors to inhibit apoptosis, maintaining continued proliferation of malignant cells in a low-androgen environment [15,16,17]. Mutations leading to activation of the PI3K/Akt pathway are reported in almost all advanced metastatic prostate cancer [18]. Loss of the phosphatase and tensin homolog (PTEN) phosphatase is the most common mechanism of constitutive activation of PI3K signaling in cancer cells [19], which was documented in 15–30% of primary, and over 60% of androgen-independent metastatic cancers [20,21,22,23,24]. 

Knocking out PTEN in the prostates of mice can trigger the development of prostate cancer [25]. Constitutive activation leads to “addiction” of cancer cells to the PI3K pathway; conversely, an inhibition of the PI3K pathway sensitizes prostate cancer cells to apoptosis [14,26,27]. Understanding PI3K/Akt/ mammalian target of rapamycin (mTOR) signaling pathways, and the development of clinically useful inhibitors remain an active area of cancer research [28,29,30,31,32,33].

In 2012 [34], we reported that prodrugs containing PI3K inhibitors could be activated via peptide cleavage by prostate-specific antigen (PSA). Cheng and co-workers also reported the generation of prostate-selective PI3K inhibitors [35]. We already showed the specific inhibition of PI3K in PSA-secreting prostate cancer cells by a Mu-LEHSSKLQL-LY294002 (prodrug-LY294002) [34]. However, LY294002 inhibits PI3K at relatively high concentrations (25 µM) and is rapidly metabolized, so while its ease of synthesis made it a good choice for proof of concept, these pharmacological properties make it a poor candidate for in vivo studies.

We were confident that PSA would cleave the Mu-LEHSSKLQL peptide after glutamine (Q), thus leaving leucine (L) attached to the PI3K inhibitor [36]. Computer modeling of the interactions between PI3K inhibitors and the ATP-binding cleft of PI3K were used to identify positions at which the PSA substrate peptide could be attached, so as to prevent the linker plus leucine from diminishing PI3K inhibition. Following our review of PI3K inhibitor literature [37], we were most interested in preparing trisubstituted pyrimidines and triazines as the next test of this prodrug concept, and here, we report our work on triazines.

## 2. Results and Discussion

### 2.1. Chemistry

**Scheme 1:**
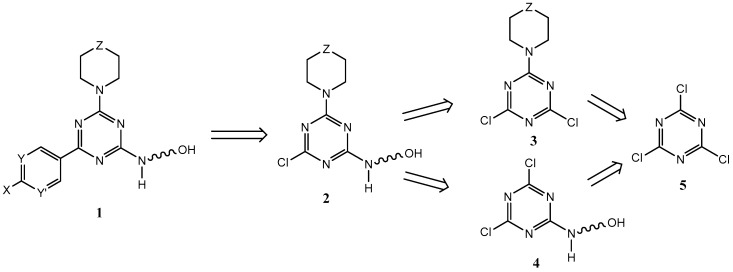
Retrosynthesis of trisubstituted triazines.

Triazines offer synthetic advantages over pyrimidines in that the addition of substituents onto its core does not produce regioisomers that have to be separated. The synthesis and characterization of trisubstituted triazines and pyrimidines remains an active area of PI3K inhibition research [38,39,40,41,42,43,44,45,46,47]. We focused most of our synthetic efforts on trisubstituted triazines (Scheme 1; **1**) which contain (1) a linker group terminating in a primary alcohol which can be used as a peptide linkage point, (2) a secondary amine (largely morpholine due to its known importance [47]), and (3) a hydrogen-bonding aromatic or heteroaromatic group. 

The triazine and pyrimidine cores offered simplicity in synthesis coupled with good PI3K inhibition activity [37,48,49,50,51,52,53,54,55,56,57,58,59]. The triazines (**1**, Scheme 1) present no regiochemical addition problems, and the trichlorotriazine (cyanuric chloride) starting material (**5**, Scheme 1) was less expensive than the 2,4,6-trichloropyrimidine; as such, we focused our efforts on the triazines. Modeling, as previously described [34,53,55,58,59], indicated that compounds containing the pyrimidine or triazine core which exhibit K_i_s < 1 µm and good water solubility and stability often contained a morpholine substituent, as well as an aromatic or heteroaromatic ring containing a hydrogen-bond donor. The third and final position on the pyrimidine/triazine core was left to be the attachment point for a functional group capable of serving as the link to the PSA cleavable peptide sequence (Mu-LEHSSKLQL). Our initial work with LY 294002 analogs [34] used linkers that terminated in OH or NH_2_ groups; so, we chose those types of linkers again. In addition to this linker, we were also interested in having a functional group in one of the other two core substituents which could interact with amino acids upon binding in PI3Ks, i.e., an ATP mimic with chemical reactivity more like wortmannin. Wortmannin functions as an irreversible PI3K inhibitor via a 1,4 conjugate addition reaction (a reaction with a soft electrophile, a Michael reaction) which takes place between the Lys 833 NH_2_ and the α,β-unsaturated ester which is contained within the fused furan and lactone rings [60,61,62].

**Scheme 2:**
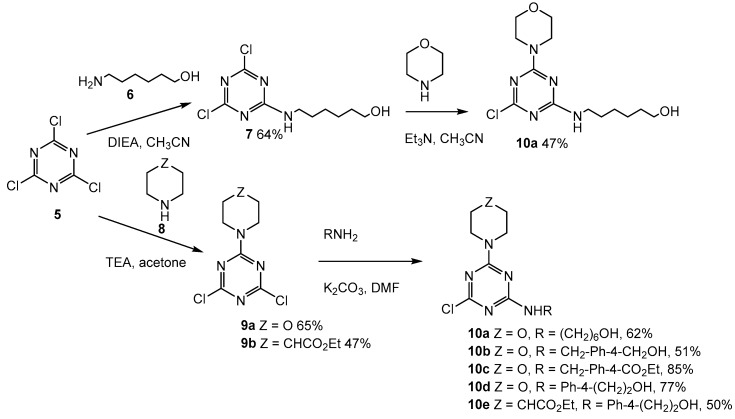
Nucleophilic aromatic substitution reactions of chlorotriazines.

Given the limiting parameters of a trisubstituted nitrogen heterocycle core containing a linker, an aromatic hydrogen-bond donor, and a heteroatom-containing secondary amine, we could, in theory, commence synthetic work by putting on any one of these three groups. In practice, we envisioned the last halogen on the heterocyclic core being replaced by a cross-coupling reaction rather than nucleophilic substitution; so, we left this for our third step. This decision meant we could investigate synthetic routes that started by adding linkers first or secondary amines (Scheme 2) first.

### 2.2. First Addition Reaction 

When we investigated the addition of aminoalcohols as the first step, we found the addition of 4-aminobenzylalcohol, 4-aminomethylbenzylalcohol, and 4-aminophenylethanol to all react sluggishly with cyanuric chloride at low temperatures (0 °C to −20 °C), and to give mixtures of mono- and di-addition products at higher temperatures (≥25 °C). Furthermore, 6-aminohexanol reacted well with cyanuric chloride to provide monosubstituted triazine (**7**, Scheme 2) with a 64% yield. We found Venkatesan’s procedure [63] of performing these reactions in acetone followed by the addition of ice water to be often preferable to other methods, since the monosubstituted triazine products precipitate under those reactions conditions. Morpholine and ethyl isonipecotate also reacted well with with cyanuric chloride under these conditions, producing **9a** and **9b** (Scheme 2).

### 2.3. Second Addition Reaction 

Compound **9a** (Scheme 2) was then treated with a variety of aromatic primary amines, **9b** (Scheme 2) was treated with 4-aminophenethylalcohol, and compound **7** (Scheme 2) was treated with morpholine. Primary amines were added to compounds **9a** and **9b** (Scheme 2) using K_2_CO_3_ in DMF with good yield. The morpholine/aminohexanol di-addition product (**10a**, Scheme 2) could be prepared as described above, starting from **9** (Scheme 2), or prepared starting from compound **7** (Scheme 2) using Et_3_N in CH_3_CN with essentially equal ease. Compounds **10a**, **10b**, and **10e** (Scheme 2) required chromatographic purification whereas **10c** and **10d** (Scheme 2) did not, which perhaps accounts for the higher isolated yields in those two cases.

### 2.4. Third Addition Reaction 

**Scheme 3:**
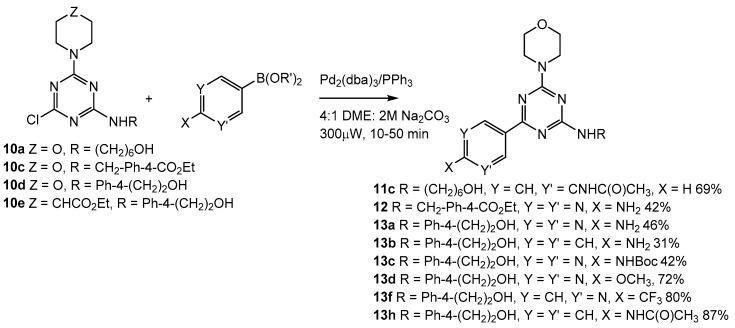
Cross coupling reactions of disubstituted chlorotriazines.

A variety of aromatic substituents were then added to these disubstituted triazines (**10a**–**d**, Scheme 2) via cross-coupling reactions, and a number of cross-coupling reaction conditions were investigated (Scheme 3). In many cases, the polarity of the final products was as much of a challenge as the cross-coupling conditions in determining isolated yields of cross-coupled products (for instance, the organic solubility of **10d** over **10b** lead us to pursue **10d** (Scheme 3)). Pd(PPh_3_)_4_ (10 mol %) was used initially as a catalyst in DMSO, THF, and DME (4:1 organic solvent, 2 M Na_2_CO_3_). DME proved superior, and we observed no product when the 2 M Na_2_CO_3_ was eliminated. Pd(dppf)Cl_2_, Pd(OAc)_2_/PPh_3_, Pd[(PtBu)_3_]_2_, and Pd_2_dba_3_/PPh_3_ were all investigated as catalysts, and the in situ-generated Pd(PPh_3_)_4_ from Pd_2_dba_3_ provided the consistently best yields of cross-coupled product. PPh_3_ proved superior as a ligand to SPhos, JohnPhos, and 9,9-dimethyl-4,5-bis(diphenylphosphino)xanthene. Polystyrene-bound PPh_3_ used with Pd_2_dba_3_ was ineffective, but polystyrene-bound Pd(PPh_3_)_4_ (PS–Pd(PPh_3_)_4_) was used successfully with **13h** (Scheme 3). In other cases, isolated yields of cross-coupled products were typically lower with PS–Pd(PPh_3_)_4_, but its use eliminated the chromatographic separation of these polar cross-coupled products from the byproduct, OPPh_3_. Our final optimized cross-coupling conditions were to use 1.5 equivalents of boronic acid or ester in 4:1 DME:2 M Na_2_CO_3_. Pd_2_dba_3_ (10 mol %) and PPh_3_ (80 mol %) were added to a sealable microwave tube, which was degassed and sealed, and irradiated 10–50 min at 300 W with a 100 °C temperature cut-off. These cross-coupling conditions never produced products from **10e** (Scheme 3) in greater than 20% isolated yield; as such, those compounds were not pursued further. 

### 2.5. Addition of Leucine to the Lead Compound (***13h***, Scheme 3)

**Scheme 4:**
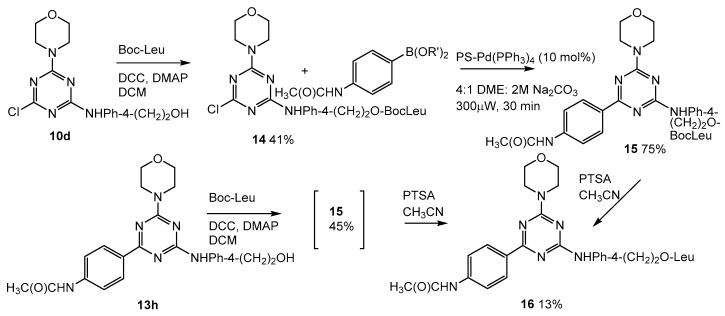
Addition of leucine to lead compound, **13h**.

All compounds **11**–**13** (Scheme 3) were screened as PI3K inhibitors, and compared directly to ZSTK 474 in this assay (see Supplementary Materials for Western blots). Trisubstituted triazine (**13h**, Scheme 2) was our most active compound as a PI3K inhibitor (see screening discussion below); therefore, we undertook its preparation where leucine was added to the primary OH in the linker group. In our earlier work [34], we showed that a PSA-cleavable peptide (Mu-LEHSSKLQ) added to produce prostate-specific prodrugs cleaved between L and Q; thus, any peptide-linked PI3K inhibitor contains a leucine residue. Two different routes to the desired compound were explored. In one case, Boc-protected leucine was added to the primary alcohol in the linker in disubstituted triazine (**10d**, Scheme 4), and then, this compound (**14**, Scheme 4) was subjected to cross-coupling conditions to produce **15** (Scheme 4), which was immediately deprotected. In an alternate route, Boc-protected leucine was added directly to **13h** (Scheme 4) to produce **15** (Scheme 4), and then, the protecting group was removed to yield **16** (Scheme 4).

### 2.6. Biological Activity

To assess the biological activity of the new PI3K inhibitor compounds, trisubstituted triazine (**13h**) and **16** (Scheme 4), the experiments were conducted in C4-2 prostate cancer cells that represent castration-resistant metastatic prostate cancer. C4-2 cells are characterized by constitutive activation of the PI3K pathway due to the loss of expression of the PI3 phosphatase, PTEN. Protein kinase B/Akt is among the best characterized downstream effectors of the PI3K pathway. Activation of PI3K leads to the accumulation of phosphatidylinositol (3,4,5)-triphosphate (PIP_3_) in the plasma membrane. PIP_3_ binds to the pleckstrin homology (PH) domains of Akt and pyruvate dehydrogenase kinases (PDKs), and recruits Akt and PDKs to the plasma membrane. This, in turn, leads to the phosphorylation of Akt at T308 by PDK1, and to phosphorylation at S473 by the rapamycin-insensitive mTOR:Rictor:Gβ complex. Thus, the phosphorylation of Akt at S473 and T308 faithfully reflect activation of the PI3K pathway in most cell lines, and are routinely used to monitor PI3K activity [64]. 

**Figure 1:**
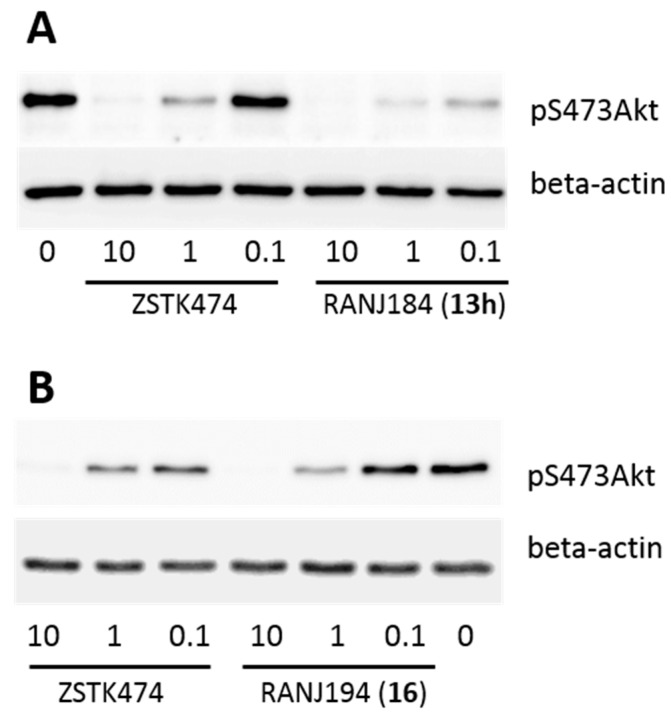
Representative western blots of S473 protein kinase B (Akt) phosphorylation in C4-2 prostate cancer cells. (**A**) C4-2 cells were treated with ZSTK474 or compound **13h** (Scheme 4; 10, 1, or 0.1 µM each). (**B**) C4-2 cells were treated with ZSTK474 or compound **16** (Scheme 4; 10, 1, or 0.1 µM each). Equal loading was controlled by probing western blot for beta-actin.

The analysis of the phosphorylation of S473 Akt and of T308 in C4-2 cells was used in this study to assess PI3K inhibition by triazine derivatives. Figure 1 shows representative western blots that illustrate the inhibition of S473 Akt phosphorylation by **13h** and by **16** (Scheme 4), which contains the leucine linker. 

Quantitation of western blots using image J software showed that respective IC_50_ values for **13h** and **16** (Scheme 4) were 6.3- and 3.6-fold, respectively, more potent compared to the well-characterized PI3K inhibitor, ZSTK474. Thus, the 50% inhibition of S473 Akt phosphorylation was observed at lower concentrations of **13h** and **16** (Scheme 4) than the 50% inhibition by ZSTK474. The comparison of phosphorylated S473 Akt (pS473 Akt) at 1 μM of ZSTK474, and compounds **13h** or **16** (Scheme 4), showed that the inhibition of S473 Akt phosphorylation by compound **13h** (Scheme 4) at 1 μM was 3.74 times more potent than inhibition by ZSTK474 at the same concentration (*p* = 0.0015); whereas inhibition by compound **16** (Scheme 4) was 2.25-fold more potent compared to ZSTK474 (*p* = 0.038). 

## 3. Materials and Methods

### 3.1. Compound Synthesis

#### 3.1.1. General Methods

Unless otherwise stated, all reactions were performed in a flame-dried round-bottom flask or a glass microwaveable tube. The flame-dried glassware was further cooled and maintained under an atmosphere of nitrogen or argon using standard Schlenk techniques before the start of the reaction. Reagents were purchased from common chemical suppliers, and were used without further purification. Analytical thin-layer chromatography (TLC) was performed on 2.5 cm × 7.5 cm Silica G TLC Plates (200-µm thickness) from Sorbtech (Norcross, GA, USA). TLC plates were pre-coated with a fluorescent indicator, and after plate development, were examined under 254-nm UV light. Flash chromatography was performed using SiliaFlash P60 230–400 mesh silica gel from Silicycle. All ^1^H- and ^13^C-NMR spectra were recorded using Bruker Avance 300 MHz or 500 MHz multinuclear spectrometers at ambient temperature. Chemical shifts were reported in parts per million (δ) relative to tetramethylsilane (TMS) or to residual resonances of the deuterated solvents. Coupling constants (*J* values) were reported in Hertz (Hz), and spin multiplicities were indicated by the following symbols: s (singlet), d (doublet), t (triplet), q (quartet), dd (double doublet), and m (multiplet). 

When stated, samples were sent off for elemental analysis to Atlantic Microlab, Inc. (Norcross, GA, USA). Samples were submitted to either the University of North Carolina at Chapel Hill’s Chemistry High Resolution Mass Spectrometry Facility or to Northwestern University’s High Resolution Mass Spectrometry Facility for HRMS analysis. HRMS analysis was also performed within Wake Forest University’s Chemistry Department using Thermo Scientific’s LTQ HRMS Orbitrap XL (Waltham, MA, USA). LC–MS analyses were performed on samples using the direct injection method for an Agilent 1100 SL meterspectro (Santa Clara, CA, USA). Lastly, TLC–MS analyses were performed using Advion (Ithaca, NY, USA) Expression-LCMS and Advion (Ithaca, NY, USA) Plate Express TLC-MS spectrometers. 

#### 3.1.2. 6-((4,6-Dichloro-1,3,5-triazin-2-yl)amino)hexan-1-ol (**7**, Scheme 2)

In a 500-mL round-bottom flask with a stir bar, cyanuric chloride (**5**, Scheme 2; 1.840 g, 10.0 mmol) was dissolved in acetonitrile (10 mL) and placed under nitrogen in an ice bath. Then, 6-amino-1-hexanol (**6**, Scheme 2; 1.880 g, 16.1 mmol) was dissolved in acetonitrile (5 mL), and *N*,*N*-diisopropylethylamine (7.18 mL, 40.0 mmol) was added. The mixture was added dropwise to the dissolved cyanuric chloride. After the dropwise addition, the ice bath was removed, and the reaction was allowed to stir for approximately one hour. The solvent was removed by rotary evaporation and high vacuum. The product was purified via column chromatography (silica, 1:1 ethyl acetate and heptanes) to give 6-((4,6-dichloro-1,3,5-triazin-2-yl)amino)hexan-1-ol (**7**, Scheme 2; 1.697 g, 6.4 mmol, 64%) R_f_ 0.40 (1:1 ethyl acetate in heptane) as a yellow waxy substance. ^1^H-NMR (300 MHz, CDCl_3_) δ 6.03 (br d, 1H), 3.66 (t, *J* = 6 Hz, 2H), 3.47 (q, *J* = 6 Hz, 2H) 1.63–1.20 (m, 9H). ^13^C-NMR (75 MHz, CDCl_3_) δ 168.7, 167.5, 163.6, 60.5, 39.2, 30.2, 26.7, 24.1, 23.

#### 3.1.3. 4-(4,6-Dichloro-1,3,5-triazin-2-yl)morpholine (**9a**, Scheme 2)

Cyanuric chloride (**5**, Scheme 2; 4.979 g, 27.0 mmol) was dissolved in acetone (50 mL) and cooled to −20 °C. Morpholine (**8**, Scheme 2; 0.7 eq., 1.654 g, 19.0 mmol) and triethylamine (0.7 eq., 1.922 g, 19.0 mmol) were mixed in an addition funnel, and added dropwise to the cooled reaction flask. The reaction stirred for 15 min before being quenched with cold water (500 mL). The solid material was allowed to settle in the flask before being filtered through a Buchner funnel, and was washed with cold methanol. The solid white material was dried overnight using a high-vacuum pump, and then, further purified using column chromatography (silica) with 20% ethyl acetate in hexanes as the eluent. White product (**9a**, Scheme 2) [65] was collected (2.903 g, 12.4 mmol, 65%). ^1^H-NMR (300 MHz, CDCl_3_) δ 3.89 (dd, *J* = 5.6, 4.0 Hz, 4H), 3.80–3.64 (m, 4H). ^13^C-NMR (75 MHz, CDCl_3_) δ 170.38, 164.04, 66.36, 44.45.

#### 3.1.4. Ethyl 1-(4,6-dichloro-1,3,5-triazin-2-yl)piperidine-4-carboxylate (**9b**, Scheme 2)

Cyanuric chloride (**5**, Scheme 2; 1.840 g, 10.0 mmol) was added to a round-bottom flask and cooled to −78 °C. In a separate container, ethyl piperidine-4-carboxylate (**8**, Scheme 2; 1 eq., 1.570 g, 10.0 mmol) and acetone (25 mL) were combined. This mixture was added dropwise to the cooled round-bottom flask using a syringe pump at a rate of 25 mL per hour. The reaction was then concentrated using rotary evaporation, before being purified using column chromatography (silica) with 1:1 ethyl acetate in hexanes as the eluent. Product (**9b**, Scheme 2) was obtained as a white solid (1.434 g, 4.7 mmol, 47%). ^1^H-NMR (300 MHz, CDCl_3_) δ 4.54 (dt, *J* = 13.6, 4.0 Hz, 2H), 4.17 (q, *J* = 7.1 Hz, 2H), 3.24 (ddd, *J* = 13.9, 11.0, 3.2 Hz, 2H), 2.63 (tt, *J* = 10.4, 4.1 Hz, 1H), 2.03 (dq, *J* = 12.6, 3.8 Hz, 2H), 1.76 (m, 2H), 1.27 (t, *J* = 7.1 Hz, 3H). ^13^C-NMR (101 MHz, CDCl_3_) δ 173.71, 170.31, 163.81, 60.81, 43.47, 40.55, 27.73, 14.20.

#### 3.1.5. 6-((4-Chloro-6-morpholino-1,3,5-triazin-2-yl)amino)hexan-1-ol (**10a**, Scheme 2)

Additionally, 6-((4,6-dichloro-1,3,5-triazin-2-yl)amino)hexan-1-ol (**7**, Scheme 2; 1.000 g, 4.0 mmol) was dissolved in acetonitrile (25 mL). Triethylamine (2.09 mL, 15.0 mmol) and morpholine (0.342 mL, 4.0 mmol) were added dropwise to the reaction mixture while stirring. The reaction was allowed to stir under nitrogen for approximately 48 h. The solvent was removed by rotary evaporation, and the crude product was purified via column chromatography (silica, 1:1 ethyl acetate and dichloromethane) to give 6-((4-chloro-6-morpholino-1,3,5-triazin-2-yl)amino)hexan-1-ol (**10a**, Scheme 2; 0.557 g, 1.76 mmol, 47%): R_f_ 0.57 (100% ethyl acetate) as a white powder. ^1^H-NMR (300 MHz, CDCl_3_) δ 5.42 (s, 1H), 4.78–4.65 (br s, 1H), 3.87–3.59 (m, 10H), 3.38 (q, *J* = 6.6 Hz, 2H), 1.67–1.49 (m, 4H), 1.40 (m, 4H). ^13^C-NMR (75 MHz, CDCl_3_) δ 168.9, 165.53, 164.22, 66.63, 62.77, 43.86, 40.84, 32.55, 29.17, 26.60, 25.43. ESI MS calculated for C_13_H_22_N_5_O_2_Cl 315.8; found 316.2. HRMS [M + H]^+^ calculated for C_13_H_23_N_5_O_2_Cl 316.1540; found 316.1526.

#### 3.1.6. (4-(((4-Chloro-6-morpholino-1,3,5-triazin-2-yl)amino)methyl)phenyl)methanol (**10b**, Scheme 2)

Furthermore, 4-(4,6-dichloro-1,3,5-triazin-2-yl)morpholine (**9a**, Scheme 2; 0.588 g, 2.5 mmol), (4-(aminomethyl)phenyl)methanol (1 eq., 0.343 g, 2.5 mmol), and K_2_CO_3_ (1 eq., 0.343 g, 2.5 mmol) were combined in a flask. The solid material was dissolved in DMF (10 mL), and was stirred for 72 h at room temperature (heated if necessary on the last day). Brine (100 mL) was added to the flask and the product was precipitated. The solid was filtered and dried overnight under high vacuum. The product was purified using column chromatography (silica) with 50% ethyl acetate in hexanes as the eluent. White product (**10b**, Scheme 2) was produced (0.428 g, 1.28 mmol, 51%). ^1^H-NMR (300 MHz, CDCl_3_) δ 7.29 (m, 4H), 5.92 (s, 1H), 4.69 (s, 2H), 4.57 (d, *J* = 6.1 Hz, 2H), 3.79 (m, 4H), 3.69 (t, *J* = 4.6 Hz, 4H), 1.88 (br s, 1H). ^13^C-NMR (101 MHz, DMSO-*d*_6_) δ 169.35, 168.74, 166.01, 165.66, 164.46, 164.32, 141.59, 137.98, 137.93, 127.65, 127.30, 126.91, 126.87, 66.16, 63.15, 44.00, 43.95. Most ^13^C-NMR resonances show evidence of two conformers due to hindered rotation around the C–NHR bond.

#### 3.1.7. Methyl 4-(((4-chloro-6-morpholino-1,3,5-triazin-2-yl)amino)methyl)benzoate (**10c**, Scheme 2) 

Moreover, 4-(4,6-dichloro-1,3,5-triazin-2-yl)morpholine (**9a**, Scheme 2; 0.825 g, 3.5 mmol), potassium carbonate (1 eq., 0.500 g, 3.5 mmol), and anhydrous DMF (20 mL) were combined and stirred at room temperature. A solution of methyl 4-(aminomethyl)benzoate (1 eq., 0.580 g, 3.5 mmol) in anhydrous DMF (5 mL) was added dropwise, and was then stirred for 72 h at room temperature. The reaction was poured into water (500 mL), and the precipitate was filtered using a Buchner funnel, washed with 20 mL cold methanol, and dried overnight. White product (**10c**, Scheme 2) was obtained (1.082 g, 2.98 mmol, 85%). Elemental analysis calculated for C_16_H_18_N_5_O_3_Cl: C, 52.82 (found 52.58); H, 4.99 (found 5.01). ^1^H-NMR (300 MHz, CDCl_3_) δ 8.01 (d, *J* = 8 Hz, 2H), 7.37 (d, *J* = 8.0 Hz, 2H), 4.66 (d, *J* = 6.1 Hz, 2H), 3.93 (s, 3H), 3.81–3.70 (m, 9H). ^13^C-NMR (75 MHz, CDCl_3_) δ 165.09, 143.39, 129.92, 127.47, 127.06, 76.72, 66.59, 66.42, 52.14, 44.63, 44.07. HRMS [M + H]^+^ calculated for C_16_H_19_N_5_O_3_Cl: 364.1176; observed 364.1171. 

#### 3.1.8. 2-(4-((4-Chloro-6-morpholino-1,3,5-triazin-2-yl)amino)phenyl)ethan-1-ol (**10d**, Scheme 2)

Subsequently, 4-(4,6-dichloro-1,3,5-triazin-2-yl)morpholine (**9a**, Scheme 2; 3.393 g, 14.4 mmol), 4-aminophenethyl alcohol (1 eq., 1.980 g, 14.4 mmol), and potassium carbonate (1 eq., 1.980 g, 14.4 mmol) were combined. The solid material was dissolved in anhydrous DMF (90 mL), and was stirred for 72 h at room temperature (monitored via TLC). The reaction contents were added into a separatory funnel with 1:1 brine and ethyl acetate, and were extracted twice (2 × 100 mL). The organic layer was dried with sodium sulfate, filtered, and concentrated using rotary evaporation and high vacuum (no further purification needed). White product (**10d**, Scheme 2) was obtained (3.730 g, 11.1 mmol, 77%). Elemental analysis calculated for C_15_H_18_N_5_O_2_Cl: C, 53.65 (found 53.45); H, 5.40 (found 5.44). ^1^H-NMR (300 MHz, DMSO-*d*_6_) δ 10.05 (s, 1H), 7.53 (d, *J* = 8.1 Hz, 2H), 7.16 (d, *J* = 8.1 Hz, 2H), 4.60 (s, 1H), 3.71–3.53 (m, 10H), 2.68 (t, *J* = 7.1 Hz, 2H), 2.45 (s, 1H). ^13^C-NMR (75 MHz, DMSO-*d*_6_) δ 136.42, 134.34, 128.94, 120.08, 65.67, 62.16, 43.66, 38.40. HRMS [M + H]^+^ calculated for C_15_H_19_ClN_5_O_2_, 336.1222; observed 336.1227. 

#### 3.1.9. Ethyl 1-(4-chloro-6-((4-(2-hydroxyethyl)phenyl)amino)-1,3,5-triazin-2-yl)piperidine-4-carboxylate (**10e**, Scheme 2) 

Ethyl 1-(4,6-dichloro-1,3,5-triazin-2-yl)piperidine-4-carboxylate (**9b**, Scheme 2; 0.687 g, 2.3 mmol), 4-aminophenethyl alcohol (1 eq., 0.309 g, 2.3 mmol), K_2_CO_3_ (1 eq., 0.311 g, 2.3 mmol), and DMF (8 mL) were combined in a round-bottom flask and stirred for 72 h at room temperature. Brine (100 mL) was added and extracted with ethyl acetate (2 × 50 mL). The organic layer was washed with additional brine solution (3 × 50 mL). The organic layer was dried with sodium sulfate, filtered, and then, concentrated via rotary evaporation. Purification involved using column chromatography (silica) with 1:1 ethyl acetate in hexanes as the eluent. Product was recovered as a white solid (0.457 g, 1.13 mmol, 50%). ^1^H-NMR (300 MHz, CDCl_3_) δ 7.45 (d, *J* = 8 Hz, 2H), 7.21 (d, *J* = 8 Hz, 2H), 7.07 (s, 1H), 4.56 (br t, 2H), 4.16 (q, *J* = 7 Hz, 2H), 3.86 (m, 2H), 3.14 (m, 2H), 2.85 (t, *J* = 6.5 Hz, 2H), 2.60 (m, 1H), 2.00 (m, 2H), 1.73 (m, 2H) 1.27 (t, *J* = 7 Hz, 3H). ^13^C-NMR (75 MHz, CDCl_3_) δ 174.17, 169.25, 164.37, 163.79, 136.28, 134.09, 129.52, 120.58, 63.62, 60.67, 43.14, 41.02, 38.57, 27.96, 27.80, 14.22 -. Elemental analysis calculated for C_19_H_24_N_5_O_3_Cl, C, 56.22 (found 55.97); H, 5.96 (found 5.96). 

#### 3.1.10. *N*-(3-(4-((6-hydroxyhexyl)amino)-6-morpholino-1,3,5-triazin-2-yl)phenyl)acetamide (**11c**, Scheme 3)

Firstly, 6-((4-chloro-6-morpholino-1,3,5-triazin-2-yl)amino)hexan-1-ol (**10a**, Scheme 3; 0.063 g, 0.2 mmol) and *N*-(3-(4,4,5,5-tetramethyl-1,3,2-dioxaborolan-2-yl)phenyl)acetamide (0.166 g, 0.4 mmol) were combined with DME (4 mL) and 2 M Na_2_CO_3_ (1 mL, 2.0 mmol). The reaction mixture was degassed with nitrogen, and PPh_3_ (0.021 g, 0.08 mmol) and Pd_2_(dba)_3_ (0.037 g, 0.04 mmol) were quickly added. The reaction mixture was placed in the CEM Discover under 300 μW and 100 °C conditions, and was set to hold for 30 minutes. After the completion time of 50 minutes, the reaction mixture was greenish black. The reaction was allowed to cool in an ice bath, was quenched with water (10 mL), and was extracted with ethyl acetate (3 × 10 mL). The organic layer was dried using NaSO_4_, and was filtered and condensed by rotary evaporation. The product was purified with column chromatography on silica gel with a 1:1 ethyl acetate and heptane solvent mobile phase to give *N*-(3-(4-((6-hydroxyhexyl)amino)-6-morpholino-1,3,5-triazin-2-yl)phenyl)acetamide (**11c**, Scheme 3; 0.057g, 0.14 mmol, 69%): R_f_ 0.04 (1:1 ethyl acetate in heptane) as a light-brown solid. ^1^H-NMR (300 MHz, DMSO-*d*_6_) δ 10.04 (s, 1H), 8.42 (s, 1H), 8.04–7.74 (m, 2H), 7.50–7.29 (m, 2H), 4.32 (t, *J* = 5.1 Hz, 1H), 3.66 (d, *J* = 4.9 Hz, 6H), 3.44–3.22 (m, 6H), 2.06 (s, *J* = 1.6 Hz, 3H), 1.36–1.27 (m, 8H). ^13^C-NMR (75 MHz, DMSO-*d*_6_) δ 169.00, 168.20, 165.59, 164.43, 139.12, 137.43, 128.28, 122.44, 121.72, 118.58, 65.88, 60.58, 43.07, 32.43, 29.24, 28.75, 26.22, 25.14, 23.87. HRMS [M + H]^+^ calculated for C_21_H_30_N_6_O_3_: 415.2379 Found: 415.2450.

#### 3.1.11. Ethyl 4-(((4-(2-aminopyrimidin-5-yl)-6-morpholino-1,3,5-triazin-2-yl)amino)methyl)benzoate (**12**, Scheme 3)

Methyl 4-(((4-chloro-6-morpholino-1,3,5-triazin-2-yl)amino)methyl)benzoate (**10c**, Scheme 3; 0.447g, 1.23 mmol), 2-aminopyrimidine-5-boronic acid pinacol ester (1.5 eq., 0.403 g, 1.83 mmol), and a 34:1 DME (17 mL):2 M Na_2_CO_3_ (500 µL) solution were added into a microwaveable tube. The mixture was degassed for 1 min before adding Pd(PPh_3_)_4_ (10 mol %). The microwave tube was heated to 100 °C for 24 h before cooling to room temperature. The microwave tube was chilled on ice before filtering the solid product (**12**, Scheme 3; 0.218 g, 0.52 mmol, 42%). ^1^H-NMR (300 MHz, CDCl_3_) δ 9.17 (s, 2H), 8.03 (d, *J* = 6.0 Hz, 2H), 7.43 (d, *J* = 6.0 Hz, 2H), 5.36 (s, 2H), 4.72 (br s, 1H), 3.93 (s, 3H), 3.87–3.75 (m, 8H). 

#### 3.1.12. 2-(4-((4-(2-Aminopyrimidin-5-yl)-6-morpholino-1,3,5-triazin-2-yl)amino)phenyl)ethan-1-ol (**13a**, Scheme 3)

Additionally, 2-(4-((4-chloro-6-morpholino-1,3,5-triazin-2-yl)amino)phenyl)ethan-1-ol (**10d**, Scheme 3; 0.200 g, 0.49 mmol), 2-aminopyrimidine-5-boronic acid pinacol ester (1.5 eq., 0.164 g, 0.74 mmol), and a 4:1 DME (8 mL) 2 M Na_2_CO_3_ (2 mL) solution were added to a microwaveable tube. The solution was degassed before adding Pd_2_(dba)_3_ (10 mol %, 0.048 g, 0.049 mmol) and PPh_3_ (80 mol %, 0.096 g, 0.38 mmol). The microwave tube was sealed and heated to 90 °C for 50 min in a microwave reactor. The microwave tube was cooled to room temperature, and was decanted into a separatory funnel containing 1:1 ether (10 mL) and water (10 mL). The aqueous phase was removed before adding 6 M HCl (10 mL) to the organic phase. The ether phase was removed, and 3 M NaOH (10 mL or enough until all HCl was quenched) was added to the aqueous phase. The product was extracted using fresh ether (10 mL), dried with sodium sulfate, filtered, and then concentrated via rotary evaporation to produce a white powder (0.089 g, 0.23 mmol, 46%). ^1^H-NMR (300 MHz, DMSO-*d*_6_) δ 9.57 (br s, 1H), 9.06 (s, 1H), 8.26–8.12 (m, 1H), 7.60 (d, *J* = 8 Hz, 2H), 7.28 (s, 1H), 7.16 (d, *J* = 8 Hz, 2H), 6.55 (m, 1H), 4.60 (br s, 1H), 3.82–3.57 (m, 8H), 3.68 (t, *J* = 7 Hz, 2H), 2.66 (t, *J* = 8 Hz, 2H).

#### 3.1.13. 2-(4-((4-(4-Aminophenyl)-6-morpholino-1,3,5-triazin-2-yl)amino)phenyl)ethan-1-ol (**13b**, Scheme 3)

Furthermore, 2-(4-((4-chloro-6-morpholino-1,3,5-triazin-2-yl)amino)phenyl)ethan-1-ol (**10d**, Scheme 3; 0.068 g, 0.20 mmol), 4-aminophenyl boronic acid pinacol ester (1.5 eq., 0.066 g, 0.30 mmol), and a 4:1 DME (4mL):2 M Na_2_CO_3_ (1 mL) solution were added to a microwaveable tube. The mixture was degassed before adding Pd_2_(dba)_3_ (10 mol %, 0.020 g, 0.020 mmol) and PPh_3_ (80 mol %, 0.040 g, 0.16 mmol). The sealed microwave tube was heated to 90 °C for 50 min in a microwave reactor. The microwave tube was cooled to room temperature and 5 mL of brine solution was added before extracting with ethyl acetate (3 × 5 mL). The organic layer was dried with sodium sulfate, filtered, and concentrated via rotary evaporation. The material was purified using column chromatography (silica) with 100% ethyl acetate as the eluent. Product was obtained a solid (0.024 g, 0.062 mmol, 31%). ^1^H-NMR (300 MHz, CDCl_3_) δ 8.24 (d, *J* = 6 Hz, 2H), 7.58 (d, *J* = 6 Hz, 2H), 7.23 (d, *J* = 6 Hz, 2H), 6.72 (d, *J* = 6 Hz, 2H), 3.95–3.63 (m, 8H), 3.87 (t, *J* = 6.5 Hz, 2H), 2.86 (t, *J* = 6.5 Hz, 2H). 

#### 3.1.14. Tert-butyl(5-(4-((4-(2-hydroxyethyl)phenyl)amino)-6-morpholino-1,3,5-triazin-2-yl)pyrimidin-2-yl)carbamate (**13c**, Scheme 3)

Moreover, 2-(4-((4-chloro-6-morpholino-1,3,5-triazin-2-yl)amino)phenyl)ethan-1-ol (**10d**, Scheme 3; 0.068 g, 0.20 mmol), tert-butyl *N*-[5-(4,4,5,5-tetramethyl-1,3,2-dioxaborolan-2-yl)pyrimidin-2-yl]carbamate (1.5 eq., 0.096 g, 0.30 mmol), and a 4:1 DME (4 mL):2 M Na_2_CO_3_ (1 mL) solution were added to a microwaveable tube. The mixture was degassed before adding Pd_2_dba_3_ (10 mol %, 0.020 g, 0.020 mmol) and PPh_3_ (80 mol %, 0.040 g, 0.16 mmol). The sealed microwave tube was heated to 90 °C for 50 min in a microwave reactor. The microwave tube was cooled to room temperature and the contents filtered through a pad of celite, and then, rinsed with ethyl acetate (~10 mL). The organic layer was dried with sodium sulfate, filtered, and concentrated via rotary evaporation. The material was then purified using column chromatography (silica) with 100% ethyl acetate as the eluent. White product was recovered as a solid (0.042 g, 0.084 mmol, 42%). Elemental analysis calculated for C_24_H_30_N_8_O_4_, C, 58.29 (found 57.46); H, 6.11 (found 6.09). ^1^H-NMR (300 MHz, CDCl_3_) δ 9.46 (s, 2H), 7.55 (d, *J* = 8.4 Hz, 2H), 7.25 (d, *J* = 8 Hz, 2H), 7.04 (br s, 1H), 3.88 (s, 9H), 3.83–3.60 (m, 8H), 3.78 (t, *J* = 6.5 Hz, 2H), 2.87 (t, *J* = 6.5 Hz, 2H), 1.56 (s, 9H). HRMS [M + H]^+^ calculated for C_24_H_31_N_8_O_4_, 495.2467; observed 495.2463. 

#### 3.1.15. 2-(4-((4-(2-Methoxypyrimidin-5-yl)-6-morpholino-1,3,5-triazin-2-yl)amino)phenyl)ethan-1-ol (**13d**, Scheme 3)

Subsequently, 2-(4-((4-chloro-6-morpholino-1,3,5-triazin-2-yl)amino)phenyl)ethan-1-ol (**10d**, Scheme 3; 0.068 g, 0.20 mmol), 2-methoxypyrimidine-5-boronic acid (1.5 eq., 0.046 g, 0.30 mmol), and a 4:1 DME (4 mL):2 M Na_2_CO_3_ (1 mL) solution were added to a microwaveable tube. The mixture was degassed before adding Pd_2_(dba)_3_ (10 mol %, 0.020 g, 0.020 mmol) and PPh_3_ (80 mol %, 0.040 g, 0.16 mmol). The sealed microwave tube was heated to 90 °C for 60 min in a microwave reactor. The reaction mixture was cooled to room temperature then filtered through a pad of celite and rinsed with ethyl acetate (~10 mL). The solution was concentrated via rotary evaporation and impurities removed through trituration with chloroform (3 × 1 mL) and acetonitrile (3 × 1 mL) to yield a white solid product (0.059 g, 0.14 mmol, 72%). ^1^H-NMR (300 MHz, CDCl_3_) δ 9.40 (s, 2H), 7.59 (d, *J* = 6 Hz, 2H), 7.23 (d, *J* = 6 Hz, 2H), 7.01 (s, 1H), 4.07 (s, 3H), 3.88 (t, *J* = 6.6 Hz, 2H), 3.90–3.63 (m, 8H), 2.87 (t, *J* = 6.5 Hz, 3H). HRMS [M + H]^+^ calculated for C_20_H_24_N_7_O_3_, 410.1940; observed 410.1941. 

#### 3.1.16. 2-(4-((4-Morpholino-6-(6-(trifluoromethyl)pyridin-3-yl)-1,3,5-triazin-2-yl)amino)phenyl)ethan-1-ol (**13f**, Scheme 3)

Then, 2-(4-((4-chloro-6-morpholino-1,3,5-triazin-2-yl)amino)phenyl)ethan-1-ol (**10d**, Scheme 3; 0.068 g, 0.20 mmol), 2-trifluoromethylpyridine-5-boronic acid (2 eq., 0.076 g, 0.40 mmol), and a 4:1 DME (4 mL):2 M Na_2_CO_3_ (1 mL) solution were added to a microwaveable tube. The mixture was degassed before adding Pd_2_dba_3_ (20 mol %, 0.037 g, 0.04 mmol) and PPh_3_ (40 mol %, 0.020 g, 0.08 mmol). The sealed microwave tube was heated to 100 °C for 20 min in a CEM Discovery microwave reactor (300 µW). The reaction was cooled to room temperature before adding water (5 mL) and extracting with ethyl acetate (4 × 5 mL). The organic layer was dried with sodium sulfate, filtered, and concentrated via rotary evaporation. Product was purified using column chromatography (silica) with 1:1 ethyl acetate in hexanes as the eluent, which was gradually changed to 100% ethyl acetate. A cream-colored product was recovered (0.071 g, 0.16 mmol, 80%). ^1^H-NMR (300 MHz, DMSO-*d*_6_) δ 9.83 (br s, 1H), 9.58 (s, 1H), 8.85 (d, *J* = 6 Hz, 1H), 8.05 (d, *J* = 6 Hz, 1H), 7.66 (d, *J* = 6 Hz, 2H), 7.19 (d, *J* = 6 Hz, 2H), 4.63 (t, *J* = 6 Hz, 2H), 3.81–3.66 (m, 8H), 2.71 (t, *J* = 6 Hz, 2H). ^13^C-NMR (75 MHz, DMSO-*d*_6_) δ 167.11, 164.29, 163.80, 149.44, 137.44, 137.10, 135.28, 133.77, 128.87, 120.60, 120.12, 65.86, 62.22, 43.43, 38.83, 38.43. HRMS [M + H]^+^ calculated for C_21_H_22_N_6_O_2_F_3_, 447.1756; observed 447.1743. 

#### 3.1.17. *N*-(4-(4-((4-(2-hydroxyethyl)phenyl)amino)-6-morpholino-1,3,5-triazin-2-yl)phenyl)acetamide (**13h**, Scheme 3)

Finally, 2-(4-((4-chloro-6-morpholino-1,3,5-triazin-2-yl)amino)phenyl)ethan-1-ol (**10d**, Scheme 3; 0.068 g, 0.20 mmol), 4-acetylaminophenyl boronic acid pinacol ester (2 eq., 0.104 g, 0.40 mmol), and a 4:1 DME (4 mL):2 M Na_2_CO_3_ (1 mL) solution were added to a microwaveable tube. The mixture degassed before adding PS–(PPh_3_)_4_–Pd (10 mol %, 0.182 g, 0.03 mmol). The sealed microwave tube was heated to 100 °C for 30 min in a CEM Discovery microwave reactor. The reaction was cooled before adding brine (5 mL) and extracting with ethyl acetate (3 × 5 mL). Saturated sodium bicarbonate solution (5 mL) was added to the aqueous layer and extracted with fresh ethyl acetate (5 mL). The organic layers were dried with magnesium sulfate, filtered, and concentrated via rotary evaporation. Product was purified using column chromatography (silica) with 5% ethanol in dichloromethane (DCM) as the eluent; gradually changed to 10% ethanol in DCM. Product was recovered as a solid (0.076 g, 0.17 mmol, 87%). ^1^H-NMR (300 MHz, DMSO-*d*_6_) δ 10.18 (s, 1H), 9.55 (s, 1H), 8.29 (d, *J* = 6 Hz, 2H), 7.76–7.60 (m, 4H), 7.17 (d, *J* = 6 Hz, 2H), 4.60 (t, *J* = 6 Hz, 1H), 3.71–3.56 (m, 8H), 2.68 (t, *J* = 6 Hz, 2H), 2.08 (s, 3H). ^13^C-NMR (75 MHz, DMSO-*d*_6_) δ 169.27, 168.61, 164.57, 164.05, 142.39, 137.58, 133.22, 130.93, 128.84, 128.76, 119.84, 118.18, 65.94, 62.26, 43.37, 24.10. HRMS [M + H]^+^ calculated C_23_H_27_N_6_O_3_, 435.2144; observed 435.2136.

#### 3.1.18. 4-((4-Chloro-6-morpholino-1,3,5-triazin-2-yl)amino)phenethyl (tert-butoxycarbonyl)leucinate (**14**, Scheme 4)

To begin, 2-(4-((4-chloro-6-morpholino-1,3,5-triazin-2-yl)amino)phenyl)ethan-1-ol (**10d**, Scheme 4; 0.068 g, 0.20 mmol), Boc-protected L-leucine (2 eq., 0.093 g, 0.40 mmol), dicyclohexylcarbodiimide (DCC; 2 eq., 0.083 g, 0.40 mmol), and dimethylaminopyridine (DMAP) (1 eq., 0.024 g, 0.20 mmol) were added to a round-bottom flask, and then, DCM (5 mL) was added before stirring overnight at room temperature (monitored via TLC analysis). The reaction was diluted with fresh DCM (15 mL) and washed with cold 1 M HCl (10 mL), saturated sodium bicarbonate solution (5 mL), and then, brine solution (5 mL). The organic layer was dried with sodium sulfate, filtered, and then, concentrated via rotary evaporation. The solid was purified by column chromatography using 1:1 ethyl acetate in hexanes are the eluent. Product (**14**, Scheme 4) was recovered as a solid (0.045 g, 0.082 mmol, 41%). ^1^H-NMR (400 MHz, CDCl_3_) δ 10.01 (s, 0H), 9.34 (d, *J* = 7.4 Hz, 1H), 7.73 (d, *J* = 8.0 Hz, 1H), 7.49–7.41 (m, 1H), 7.37 (d, *J* = 7.7 Hz, 1H), 7.20 (dd, *J* = 7.7, 5.6 Hz, 1H), 5.33 (s, 0H), 4.94–4.77 (m, 1H), 4.40–4.24 (m, 1H), 3.91 (t, *J* = 4.7 Hz, 1H), 3.87–3.76 (m, 1H), 3.79 (s, 1H), 3.74 (d, *J* = 4.8 Hz, 2H), 3.51 (s, 1H), 3.43 (s, 2H), 2.94 (td, *J* = 7.0, 3.7 Hz, 1H), 1.90 (dt, *J* = 12.4, 3.8 Hz, 2H), 1.74–1.59 (m, 2H), 1.48–1.34 (m, 9H), 1.38–1.23 (m, 1H), 1.22–1.05 (m, 2H), 0.98–0.86 (m, 6H).

#### 3.1.19. 4-((4-(4-Acetamidophenyl)-6-morpholino-1,3,5-triazin-2-yl)amino)phenethyl (tert-butoxycarbonyl)leucinate (**15**, Scheme 4)

Furthermore, 4-((4-chloro-6-morpholino-1,3,5-triazin-2-yl)amino)phenethyl (*tert*-butoxycarbonyl)leucinate (**14**, Scheme 4; 0.055 g, 0.10 mmol), 4-acetylaminophenyl boronic acid pinacol ester (2 eq., 0.052 g, 0.20 mmol), and a 4:1 DME (4 mL):2 M Na_2_CO_3_ (1 mL) solution were added to a microwaveable tube. The mixture was degassed for 15 minutes before adding PS–(PPh_3_)_4_–Pd (10 mol %, 0.182 g, 0.03 mmol). The sealed microwave tube was heated to 100 °C for 30 min in a CEM Discovery microwave reactor. The reaction was cooled before adding brine (5 mL) and extracting with ethyl acetate (3 × 5 mL). Saturated sodium bicarbonate solution (5 mL) was added to the aqueous layer and extracted with fresh ethyl acetate (5 mL). Organic layers were combined and dried with sodium sulfate, filtered, and concentrated via rotary evaporation. Product was purified using column chromatography with 1:1 ethyl acetate in hexanes as the eluent, which was gradually changed to 100% ethyl acetate. Product (**15**, Scheme 4) was recovered (0.049 g, 0.075 mmol, 75%; identical by MS comparison to **15** (Scheme 4) described below), and was immediately deprotected in a second reaction. 

#### 3.1.20. 4-((4-(4-Acetamidophenyl)-6-morpholino-1,3,5-triazin-2-yl)amino)phenethyl (tert-butoxycarbonyl)leucinate (**15**, Scheme 4)

In acetonitrile (2 mL), Boc-leucine (0.011 g, 0.049 mmol), *N*,*N*′-dicyclohexylcarbodiimide (0.013 g, 0.065 mmol), and 4-dimethylaminopyridine (one crystal) were added. To this mixture, N-(4-(4-((4-(2-hydroxyethyl)phenyl)amino)-6-morpholino-1,3,5-triazin-2-yl)phenyl)acetamide (**13h**, Scheme 4; 0.014 g, 0.032 mmol) in acetonitrile (2 mL) was added and stirred for 48 h at room temperature. After 48 h, an additional measure of Boc-leucine (0.011 g, 0.049 mmol), *N*,*N*′-dicyclohexylcarbodiimide (0.013 g, 0.065 mmol), and 4-dimethylaminopyridine (one crystal) were added and stirred for an additional 24 h at room temperature. After 24 h, the reaction was concentrated into a solid powder via rotary evaporation, and then, purified using column chromatography (1:1 ethyl acetate in hexanes). Product (**15**, Scheme 4) was recovered as a solid (0.009 g, 0.014 mmol, 43%). ESI MS M+ calculated for C_33_H_45_N_7_O_6_ 635.7; found 635.3 (100), 592.2 (10), 460.2 (10), 306.3 (34), 266.2 (22), 225.2 (10).

#### 3.1.21. 4-((4-(4-Acetamidophenyl)-6-morpholino-1,3,5-triazin-2-yl)amino)phenethyl leucinate (**16**, Scheme 4)

Additionally, 4-((4-(4-acetamidophenyl)-6-morpholino-1,3,5-triazin-2-yl) amino) phenethyl (*tert*-butoxycarbonyl) leucinate (**15**, Scheme 4; 0.046 g, 0.071 mmol), para-toluenesulfonic acid (2 eq., 0.027 g, 0.14 mmol), and acetonitrile (5 mL) were added to a round-bottom flask and stirred for 48 h at room temperature. On the second day, another equivalent of para-toluenesulfonic acid (0.014 g, 0.071 mmol) was added. The reaction stirred for another 24 h before concentrating via rotary evaporation. The contents were then dissolved in cold water (3 mL) and ethyl acetate (5 mL). The aqueous layer was removed, and saturated sodium bicarbonate (3 mL) was added to the organic layer. This aqueous layer was extracted three additional times with ethyl acetate (3 × 5mL). Organic layers were combined, dried with sodium sulfate, filtered, and then, concentrated via rotary evaporation. Column chromatography (silica) was performed using 1:1 ethyl acetate in hexanes as the eluent, which was gradually changed to 100% ethyl acetate, then 10% ethanol in ethyl acetate. Product (**16**, Scheme 4) was recovered as a solid (0.005 g, 0.009 mmol, 13%; ~90% pure) and all this material was used for screening. ESI MS M+ calculated for C_29_H_37_N_7_O_4_ 547.6; found 547.8 (100), 527.9 (15), 434.8 (30), 414.9 (50), 301.9 (25), 283.9 (10). 

### 3.2. Western Blot Analysis on Synthesized PI3K Inhibitors

#### General Methods

Media were removed and cells were washed gently twice using cold phosphate-buffered saline (PBS) on ice. Lysates were generated using cold 20 mM HEPES, 150 mM NaCl, 1 mM EDTA, 0.5% Na + deoxycholate, 1% Nonidet P-40, 1 mM DTT pH 7.4 buffer-containing protease inhibitors (10 μg/mL aprotinin, 10 μg/mL leupeptin, 10 μg/mL pepstatin, 1 mM benzamidine, and 1 mM PMSF), and phosphatase inhibitors (1 mM NaVO_4_, 50 mM β-glycerophosphate, 40 mM *p*-nitrophenylphosphate, 40 mM NaF, and 1 μg/mL microcystin). All reagents were purchased from Sigma-Aldrich (St. Louis, MO, USA). Cell lysates were clarified by centrifugation at 15,000 rpm for 15 min at 4 °C, and the supernatant was collected and the protein content measured by a Bradford assay (Bio-Rad Laboratories, Hercules, CA, USA) per the manufacturer’s directions. Proteins were separated on SDS-PAGE on 12% gels, and were transferred onto a 0.45-μm nitrocellulose membrane (PerkinElmer, Waltham, MA, USA). Western blotting was performed with the Odyssey CLx Infrared Imaging System (Li-Cor Biosciences, Lincoln, NE, USA) per the manufacturer’s instructions. Rabbit polyclonal phosphorylated Akt (Ser473; D9E) XP, phosphorylated Akt (Thr308; D25E6) XP, and anti-Akt were purchased from Cell Signaling Technology (Beverly, MA, USA). Mouse monoclonal anti-β-Actin was purchased from Sigma-Aldrich. Secondary goat anti-mouse IRDye 680 and goat-anti rabbit IRDye 800 were both purchased from Li-Cor Biosciences. Protein bands were quantified using ImageJ software (National Institutes of Health, USA). Rabbit mAb #4060, Ref: 08/2015, Lot:19 (Cell Signaling). ZSTK474 is an ATP-competitive inhibitor of class I PI3K isoforms with an IC_50_ value of 37 nM in a cell-free assay. We used this PI3K inhibitor as a control when testing our synthesized compounds. 

## 4. Conclusions

In conclusion, we conducted the synthesis and pilot characterization of a new trisubstituted triazine PI3K inhibitor with increased potency at 1 M (2–4-fold) compared to the well-characterized PI3K inhibitor, ZSTK474. At concentrations lower than 1 M, our data cannot be unambiguously interpreted, and additional experiments are needed in the future. We also demonstrated that this new compound can be used to produce a latent prodrug selectively activated in prostate tumors that secretes the prostate-specific antigen (PSA) protease. Thus, attachment of a leucine linker did not prevent the inhibition of Akt phosphorylation, and hence, the inhibition of PI3K by the modified inhibitor. The leucine linker remains on the prodrug after cleavage by PSA of an inhibitor peptide, preventing penetration of the inactive prodrug into target cells. Future experiments will examine the toxicity profile of the new PI3K inhibitor, and will determine if prodrugs based on this compound would provide more potent inhibition of PI3K in prostate tumors that secrete PSA compared to currently used PI3K inhibitors. 

## Supplementary Materials

Supplementary materials include spectral data for new compounds, and can be accessed at.
