# Supplementary material for: Synthesis and PI 3-Kinase Inhibition Activity of Some Novel 2,4,6-Trisubstituted 1,3,5-Triazines
Source: Molecules. 2018 Jul 4;23(7):1628. doi: 10.3390/molecules23071628 (PMC6100378; doi:10.3390/molecules23071628)

Synthesis and PI3 Kinase Inhibition Activity of Some Novel 2,4,6-Trisubstituted 1,3,5-Triazines

Ronald A. Nelson, Jr.[2], Taylor Schronce[2], Yue Huang[1], Arwa Alghuneim[3], Lamya Al Fuhaid[3], Alanoud Albugumi[3], George Kulik[1,3]* and Mark E. Welker[2]*

[1]Department of Cancer Biology and Comprehensive Cancer Center, Wake Forest School of Medicine, Medical Center Blvd., Winston-Salem, NC 27157, USA

[2]Department of Chemistry, Wake Forest University, Winston-Salem, NC 27109, USA

[3] Life Sciences program, College of Science, Alfaisal University, Riyadh 11533, Saudi Arabia.

*To whom correspondence should be addressed.

George Kulik, DVM, PhD. Department of Cancer Biology and Comprehensive Cancer Center Wake Forest University School of Medicine, Medical Center Blvd., Winston-Salem, NC 27127-1083, USA. Tel.: 336-713-7650. E-mail: gkulik@wakehealth.edu

Mark E Welker, PhD. Department of Chemistry, Wake Forest University, P.O. Box 7486, Winston-Salem, NC 27109, USA. Tel.: 336-758-5758. E-mail: welker@wfu.edu

Supplementary Material: Spectral Data for New Compounds Reported Follows.

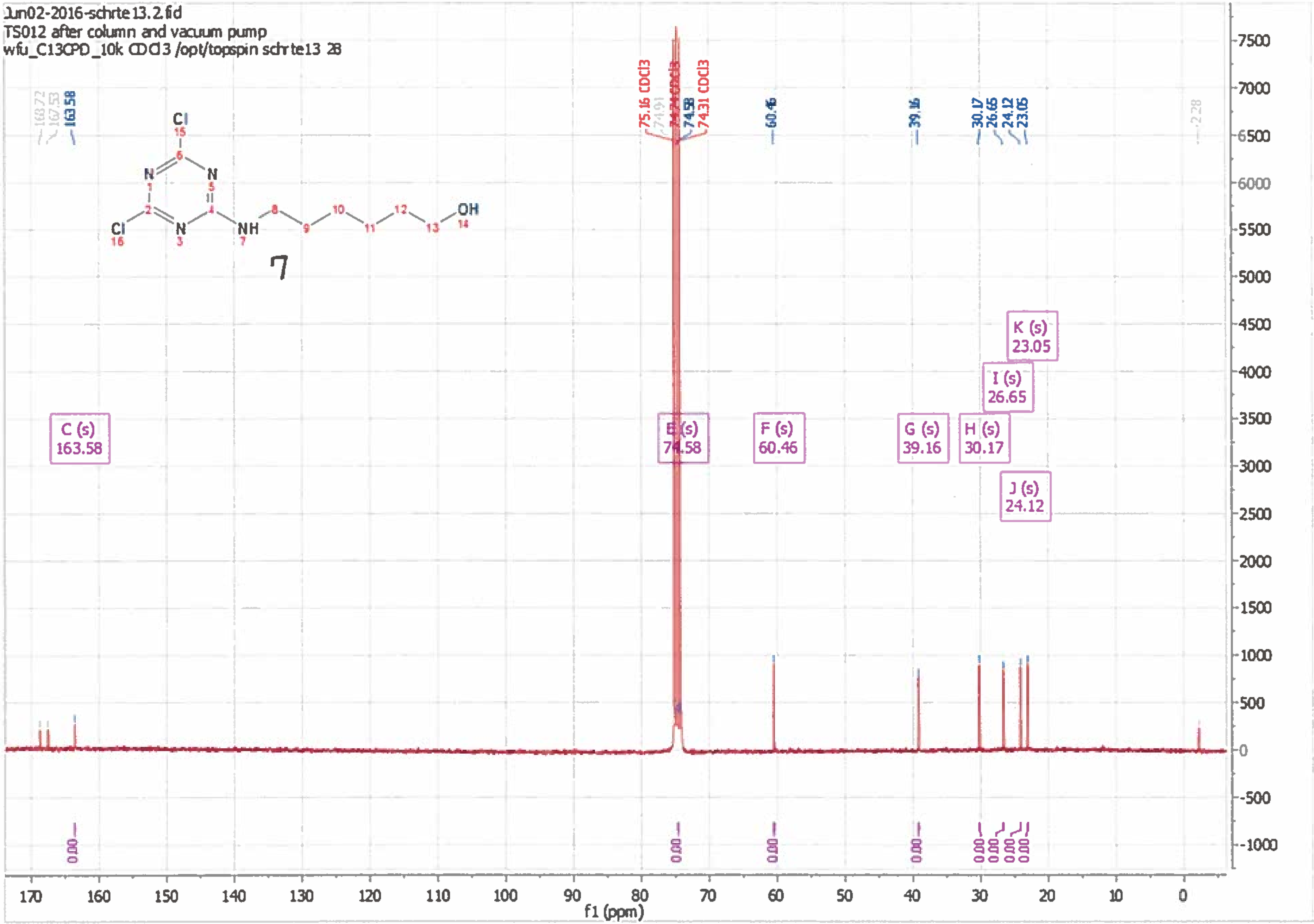

1un02-2016-schrte13.2.fid
TS012 after column and vacuum pump
wfu_C13CPD_10k CDCl3 /opt/topspin schrte13 28
168.72
167.53
163.58
75.16 CDCl3
74.91
74.74 CDCl3
74.58
74.31 CDCl3
60.46
39.16
30.17
26.65
24.12
23.05
2.28
7
C (s) 163.58
E (s) 74.58
F (s) 60.46
G (s) 39.16
H (s) 30.17
I (s) 26.65
J (s) 24.12
K (s) 23.05
0.00
f1 (ppm)

4-(4,6-dichloro-1,3,5-triazin-2-yl)morpholine, 9a.

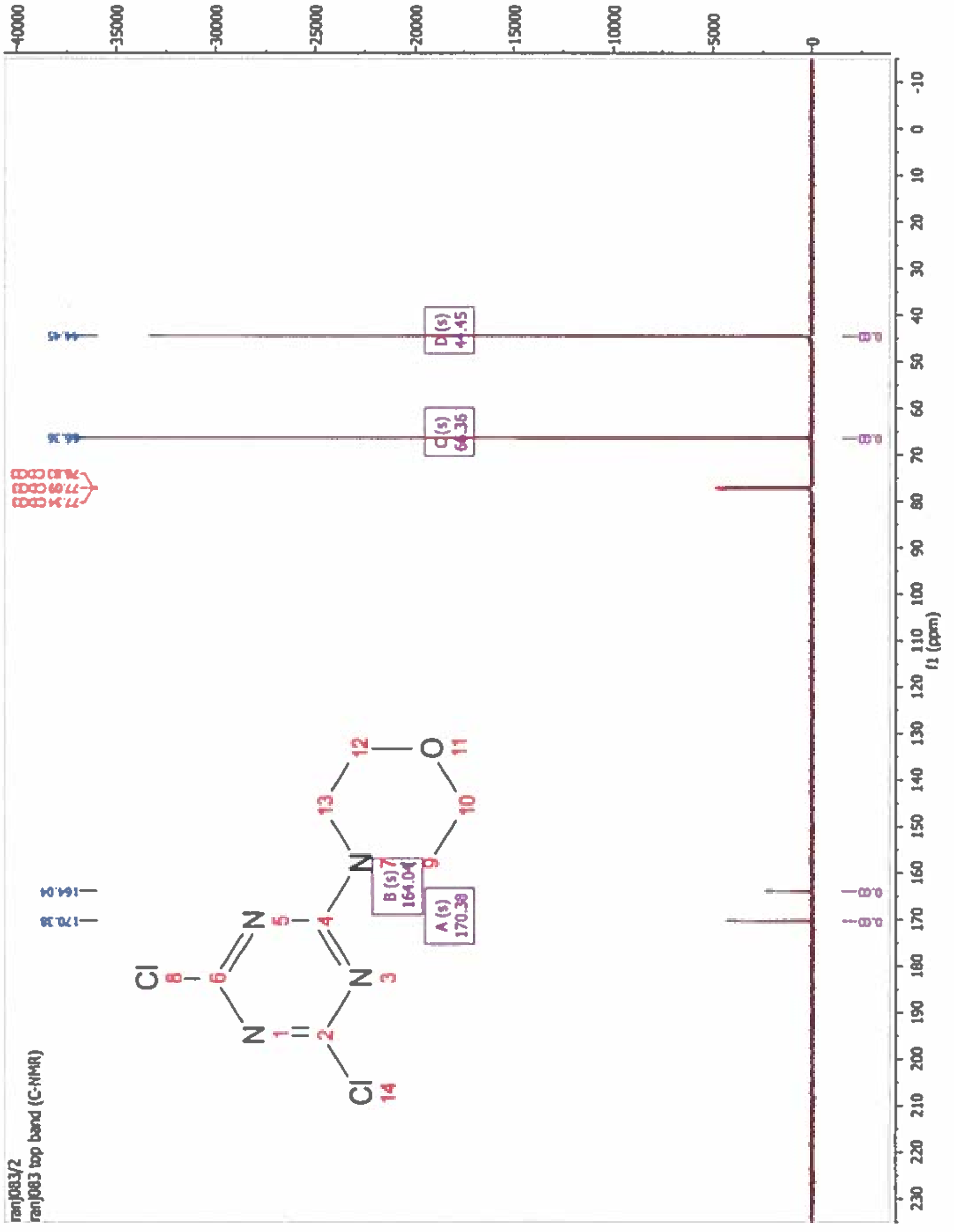

ethyl 1-(4-chloro-6-((4-(2-hydroxyethyl)phenyl)amino)-1,3,5-triazin-2-yl)piperidine-4-carboxylate, 9b.

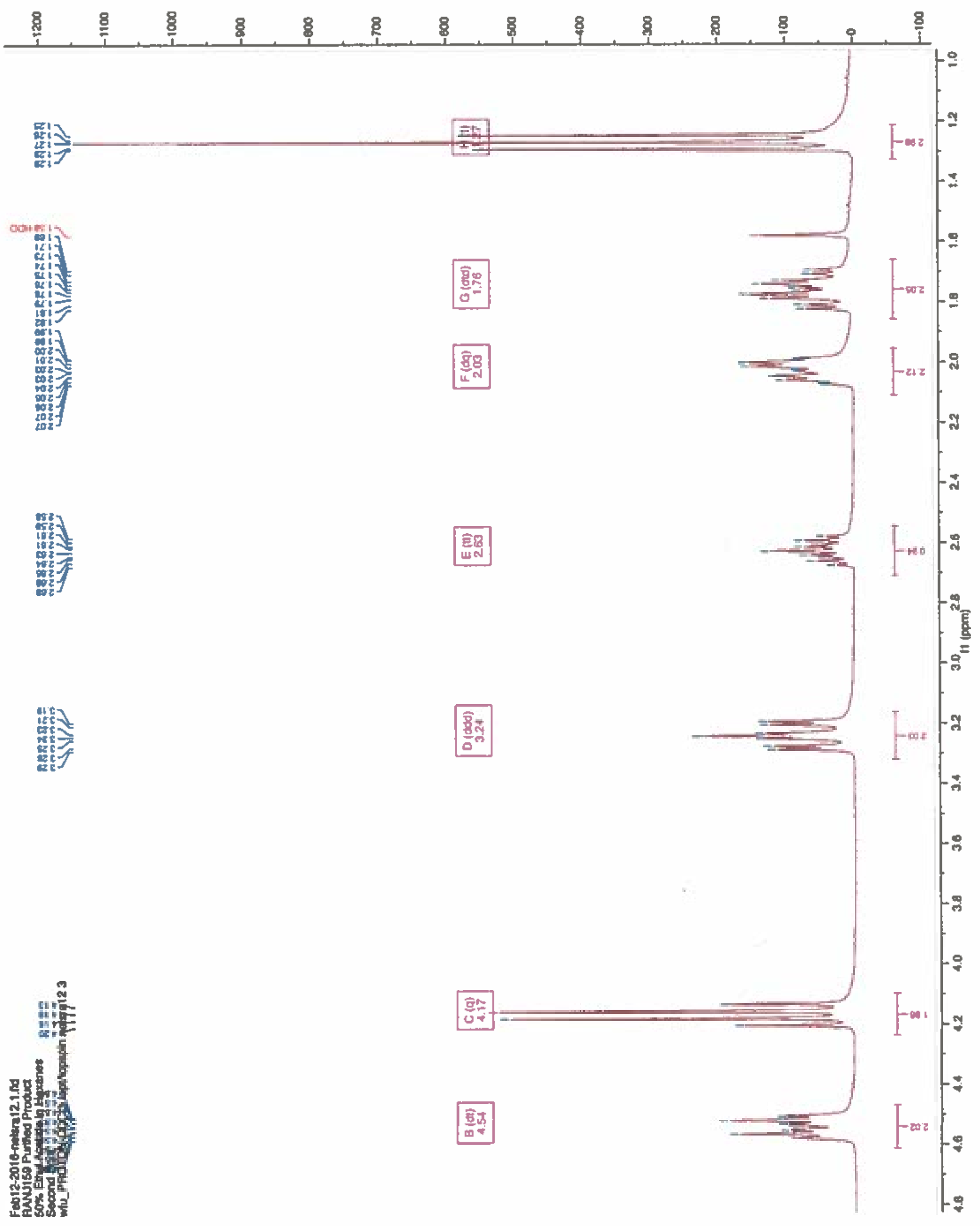

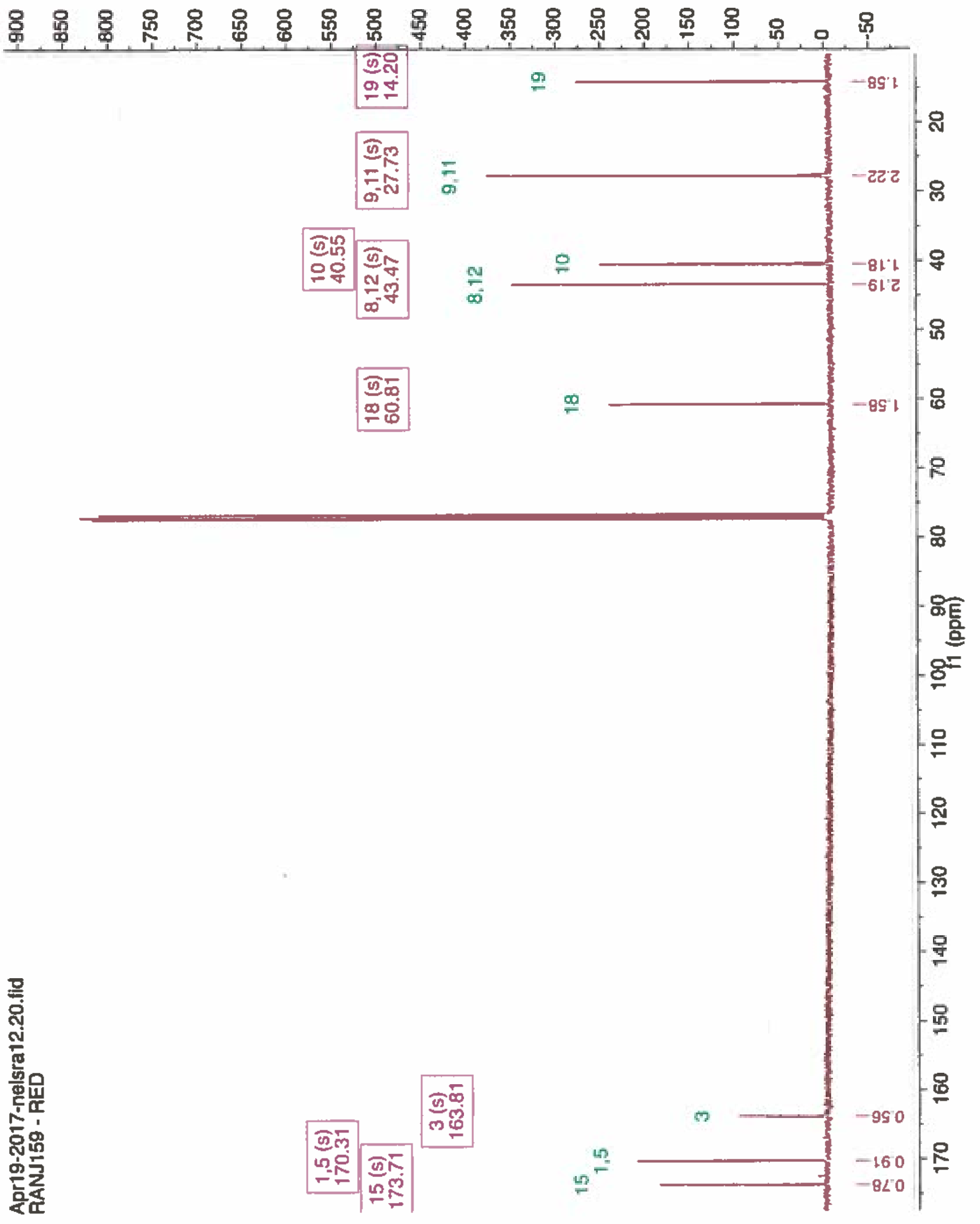
Apr19-2017-nelsra12.20.fid
RANJ159 - RED
19 (s) 14.20
9,11 (s) 27.73
10 (s) 40.55
8,12 (s) 43.47
18 (s) 60.81
3 (s) 163.81
1,5 (s) 170.31
15 (s) 173.71
19
9,11
10
8,12
18
3
1,5
15
1.58
2.22
1.18
2.19
1.58
0.56
0.91
0.78
f1 (ppm)

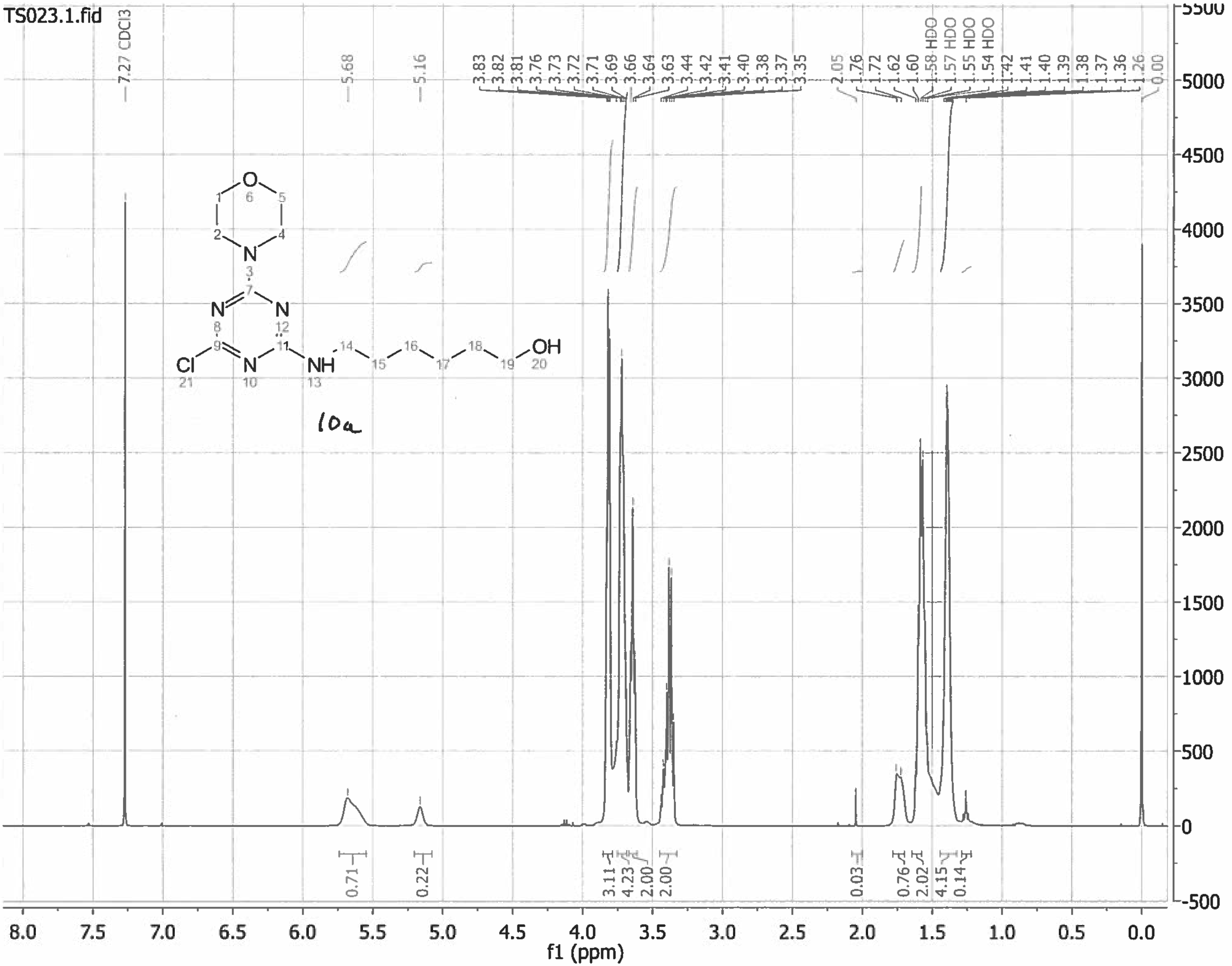

TS023.1.fid
7.27 CDCl3
5.68
5.16
3.83 3.82 3.81 3.76 3.73 3.72 3.71 3.69 3.66 3.64 3.63 3.44 3.42 3.41 3.40 3.38 3.37 3.35
2.05 1.76 1.72 1.62 1.60 1.58 HDO 1.57 HDO 1.55 HDO 1.54 HDO 1.42 1.41 1.40 1.39 1.38 1.37 1.36 1.26 0.00
O
N
N
N
N
Cl
NH
OH
10a
0.71
0.22
3.11
4.23
2.00
2.00
0.03
0.76
2.02
4.15
0.14
f1 (ppm)

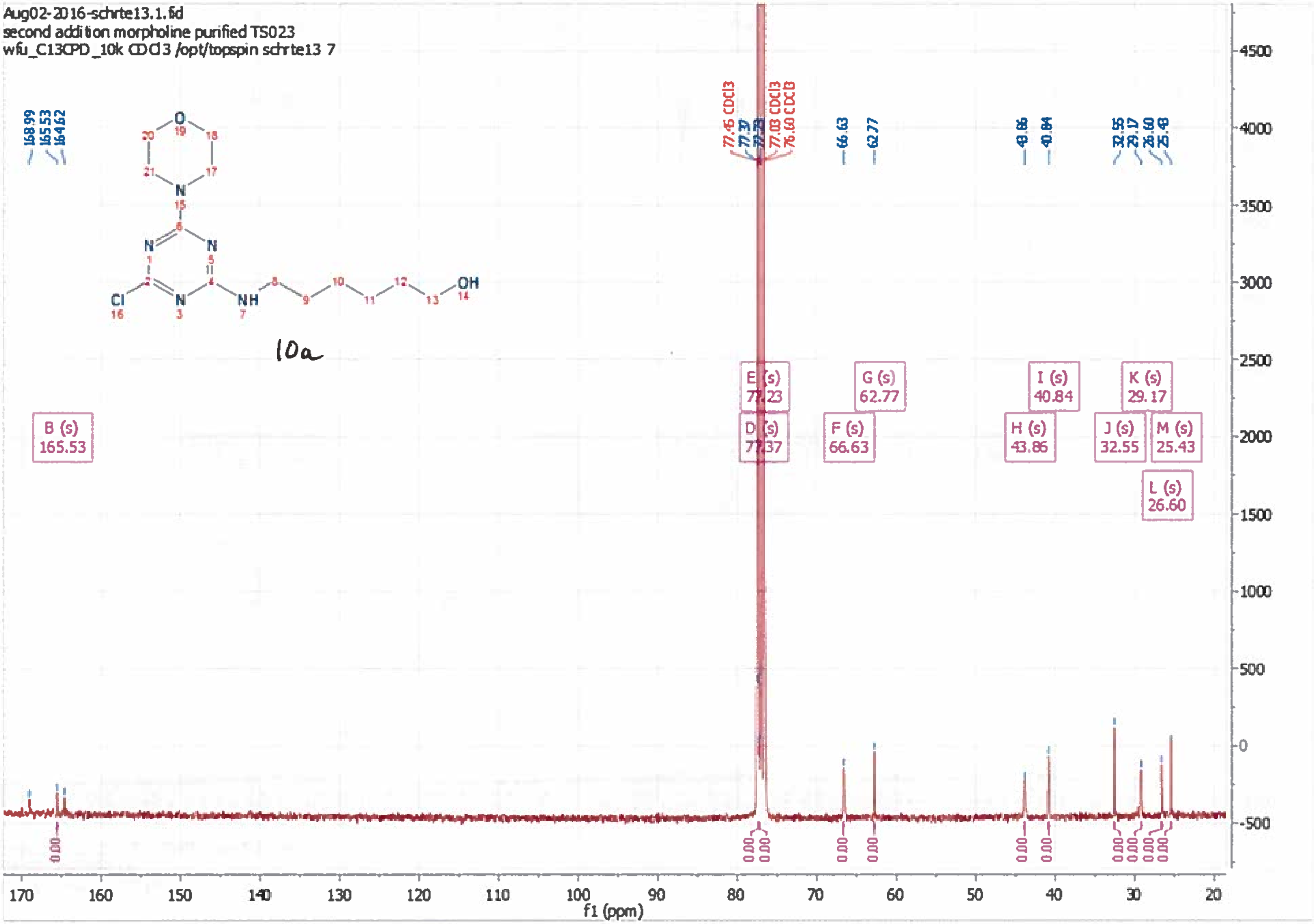

Aug02-2016-schrte13.1.fid
second addition morpholine purified TS023
wfu_C13CPD_10k CDCl3 /opt/topspin schrte13 7
10a
168.99
165.53
164.62
77.45 CDCl3
77.37
77.23
77.03 CDCl3
76.60 CDCl3
66.63
62.77
43.86
40.84
32.55
29.17
26.60
25.43
B (s) 165.53
D (s) 77.37
E (s) 77.23
F (s) 66.63
G (s) 62.77
H (s) 43.86
I (s) 40.84
J (s) 32.55
K (s) 29.17
L (s) 26.60
M (s) 25.43
f1 (ppm)

(4-(((4-chloro-6-morpholino-1,3,5-triazin-2-yl)amino)methyl)phenyl)methanol, 10b.

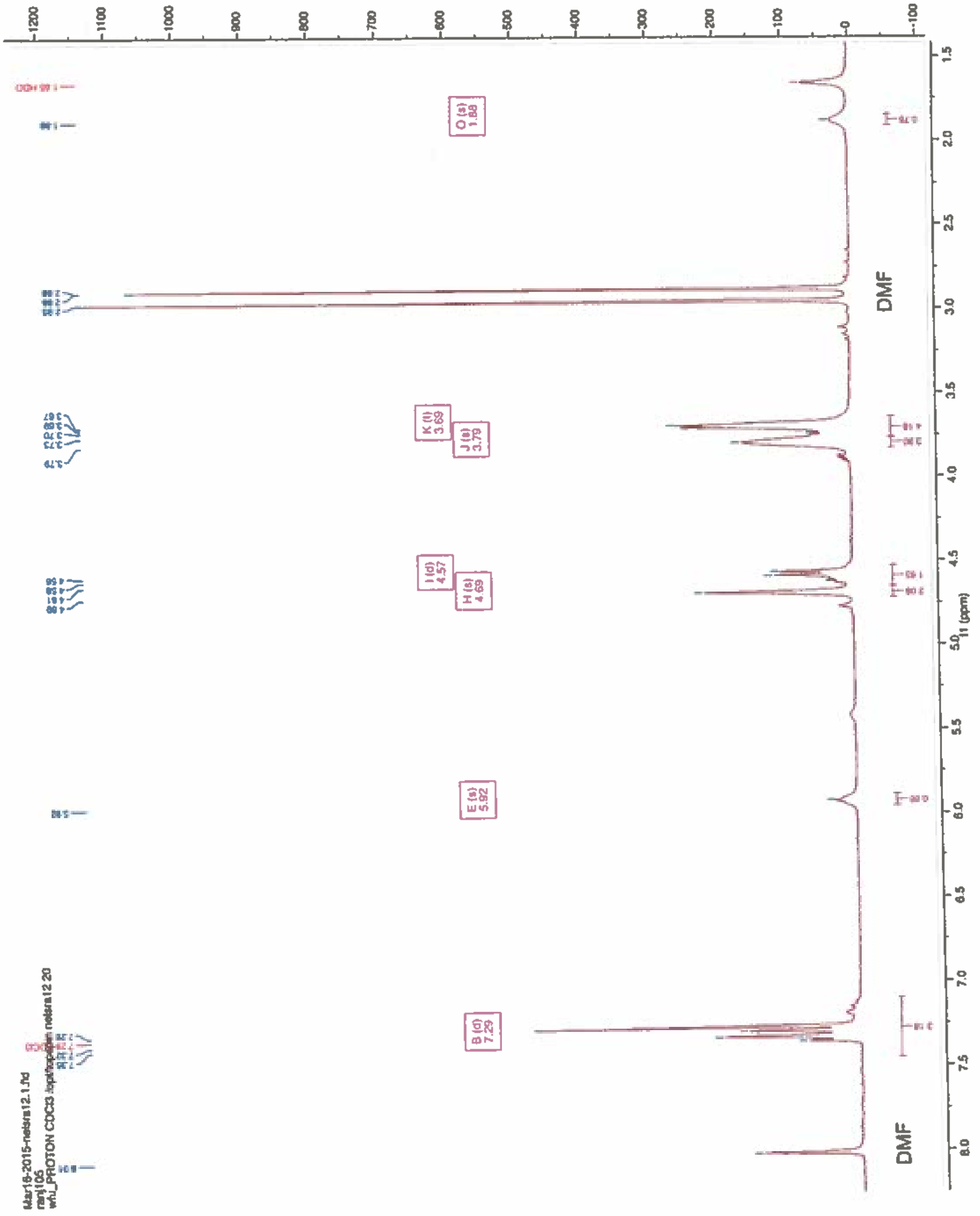

methyl 4-(((4-chloro-6-morpholino-1,3,5-triazin-2-yl)amino)methyl)benzoate, 10c.

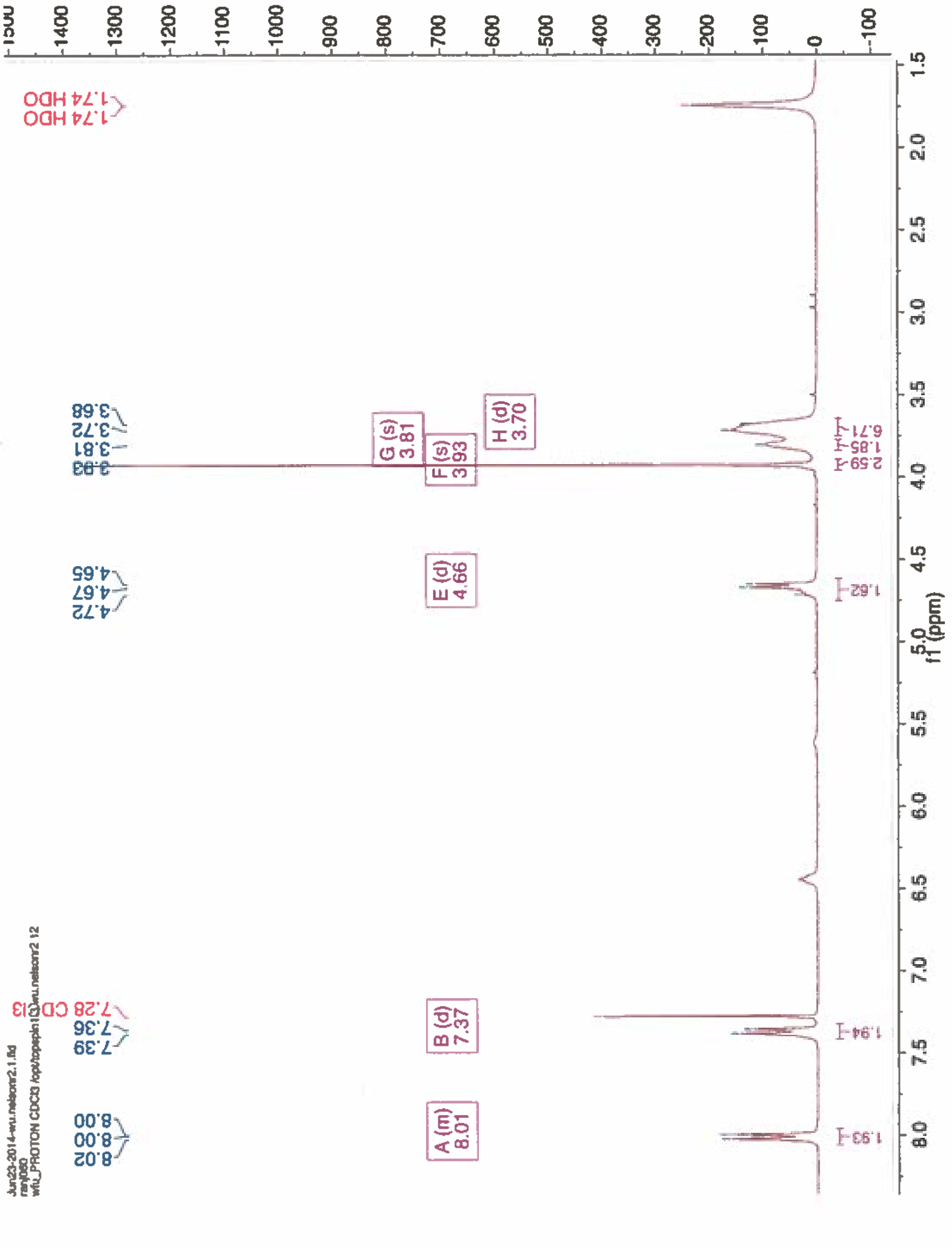

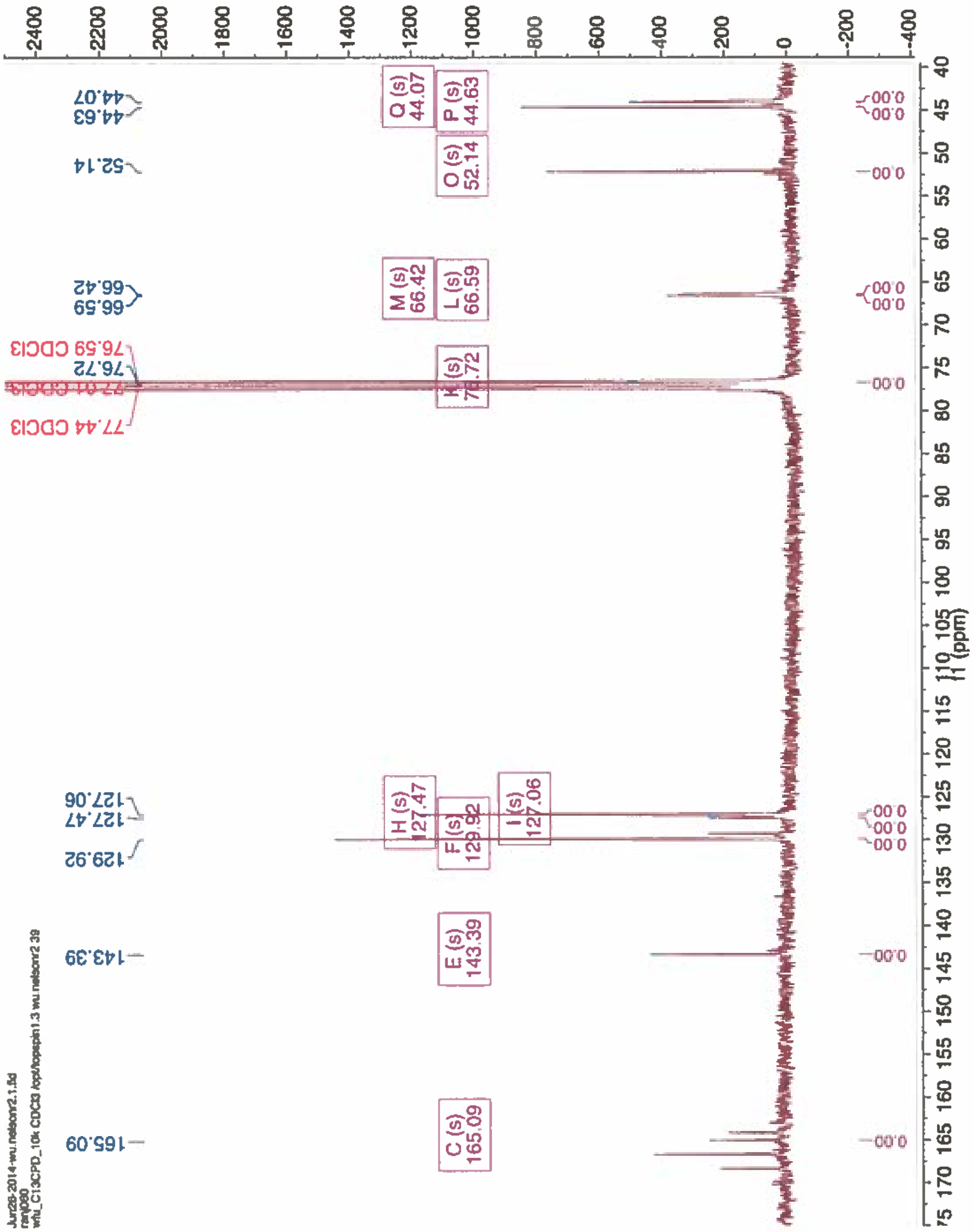

44.07
44.63
52.14
66.42
66.59
76.59 CDCl3
76.72
77.01 CDCl3
77.44 CDCl3
127.06
127.47
129.92
143.39
165.09
f1 (ppm)

2-(4-((4-chloro-6-morpholino-1,3,5-triazin-2-yl)amino)phenyl)ethan-1-ol, 10d.

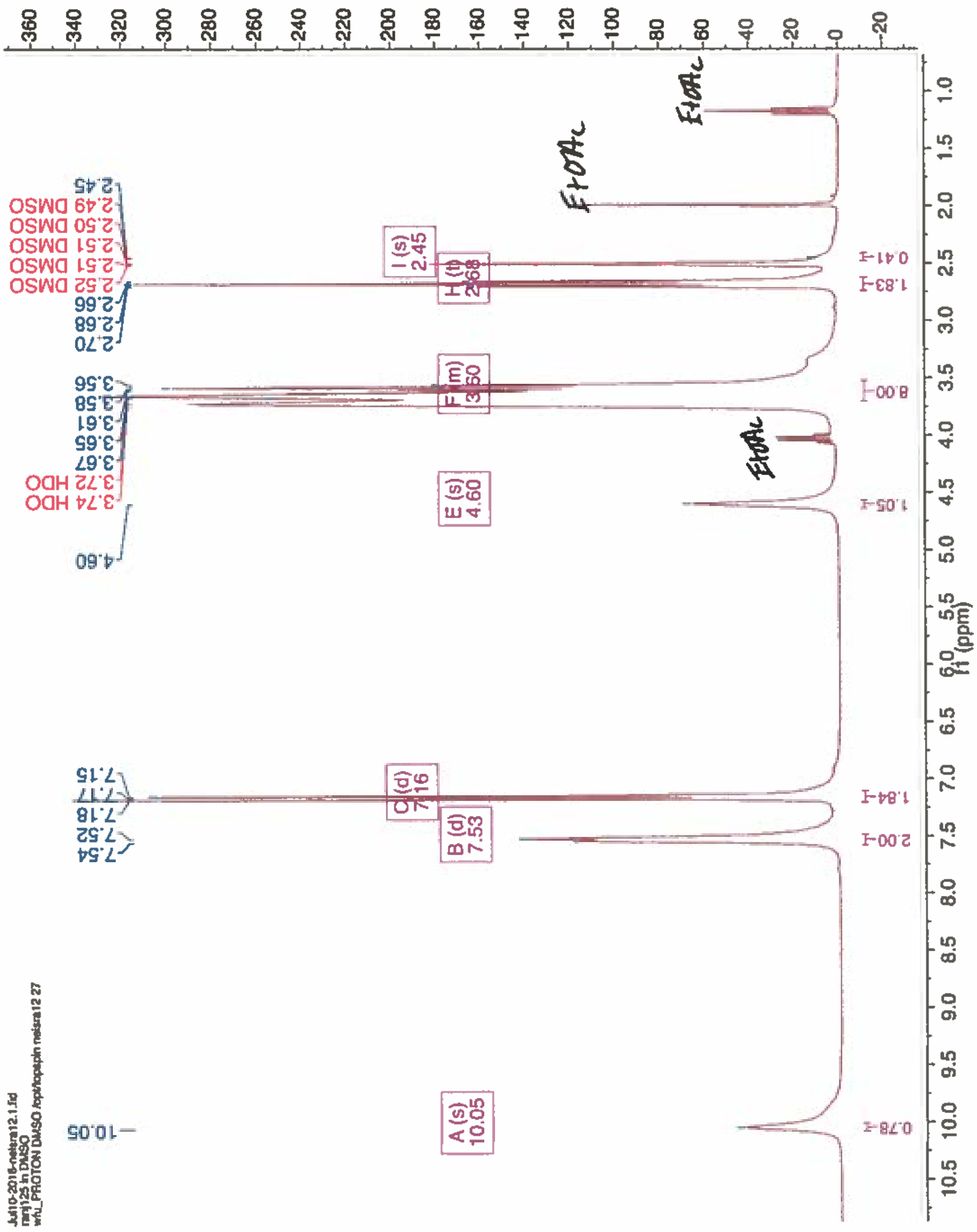

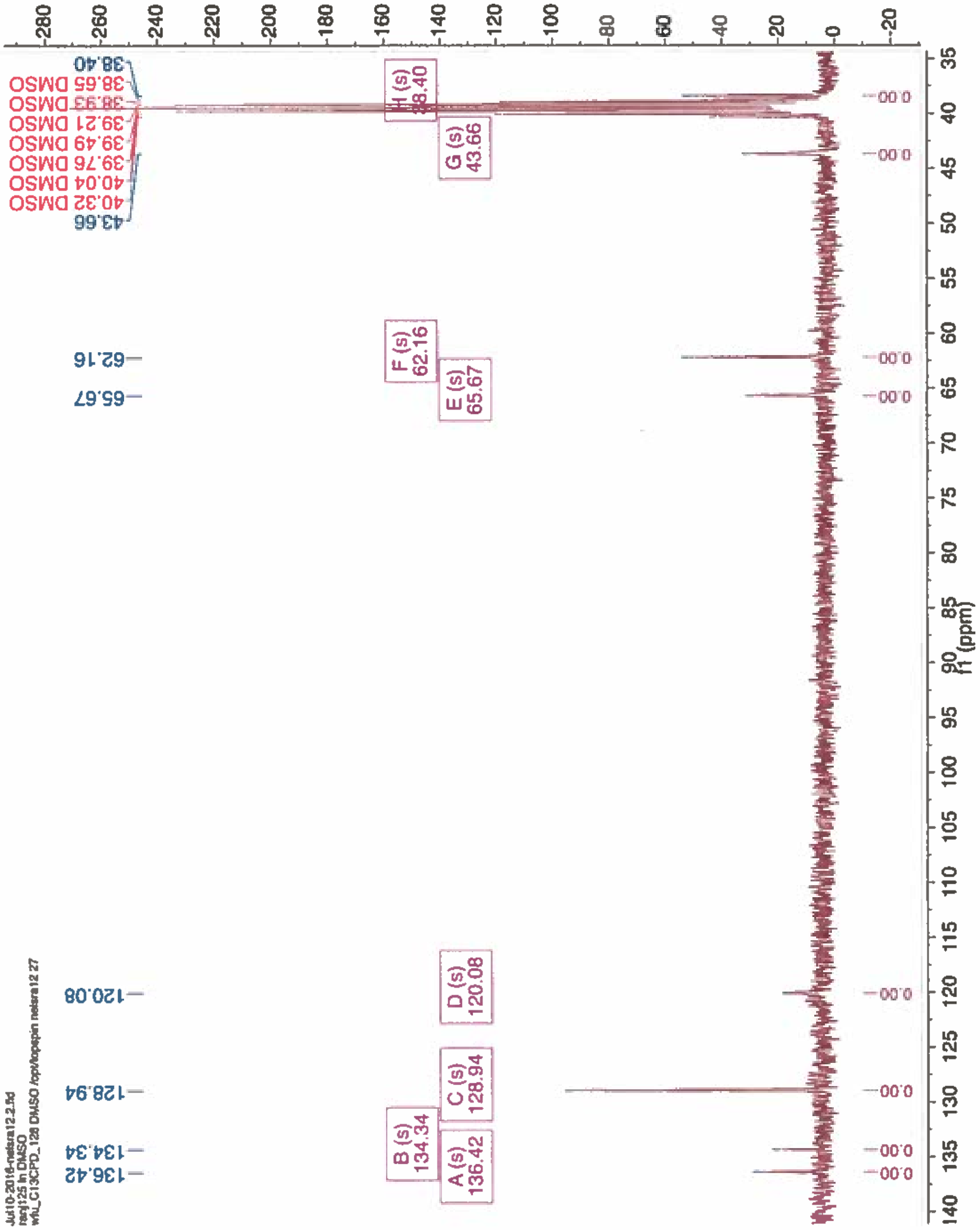
Jul10-2016-netsra12.2.fid
f1 (ppm)
136.42
134.34
128.94
120.08
65.67
62.16
43.66
40.32 DMSO
40.04 DMSO
39.76 DMSO
39.49 DMSO
39.21 DMSO
38.93 DMSO
38.65 DMSO
38.40
A (s) 136.42
B (s) 134.34
C (s) 128.94
D (s) 120.08
E (s) 65.67
F (s) 62.16
G (s) 43.66
H (s) 38.40

ethyl 1-(4-chloro-6-((4-(2-hydroxyethyl)phenyl)amino)-1,3,5-triazin-2-yl)piperidine-4-carboxylate, 10e.

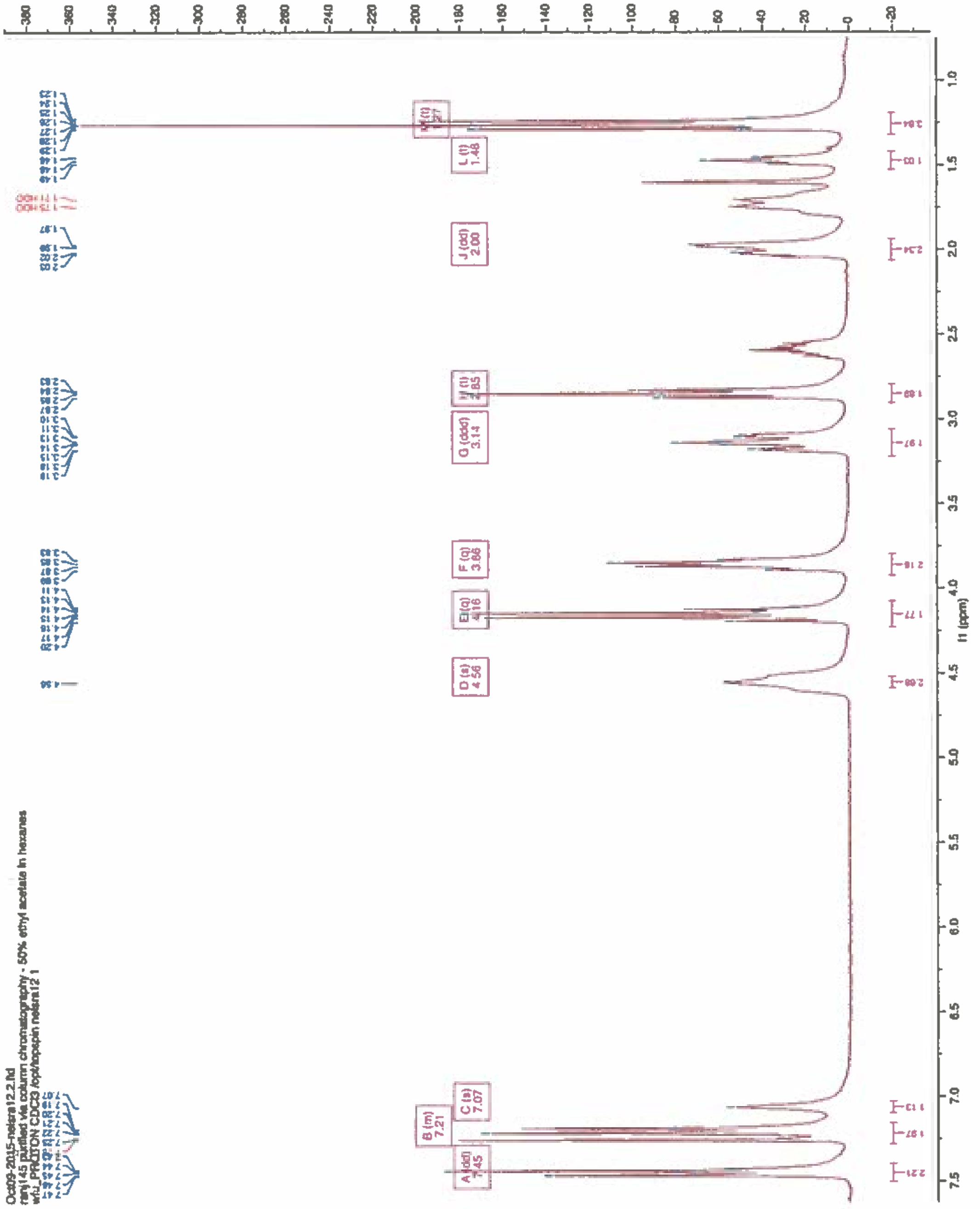

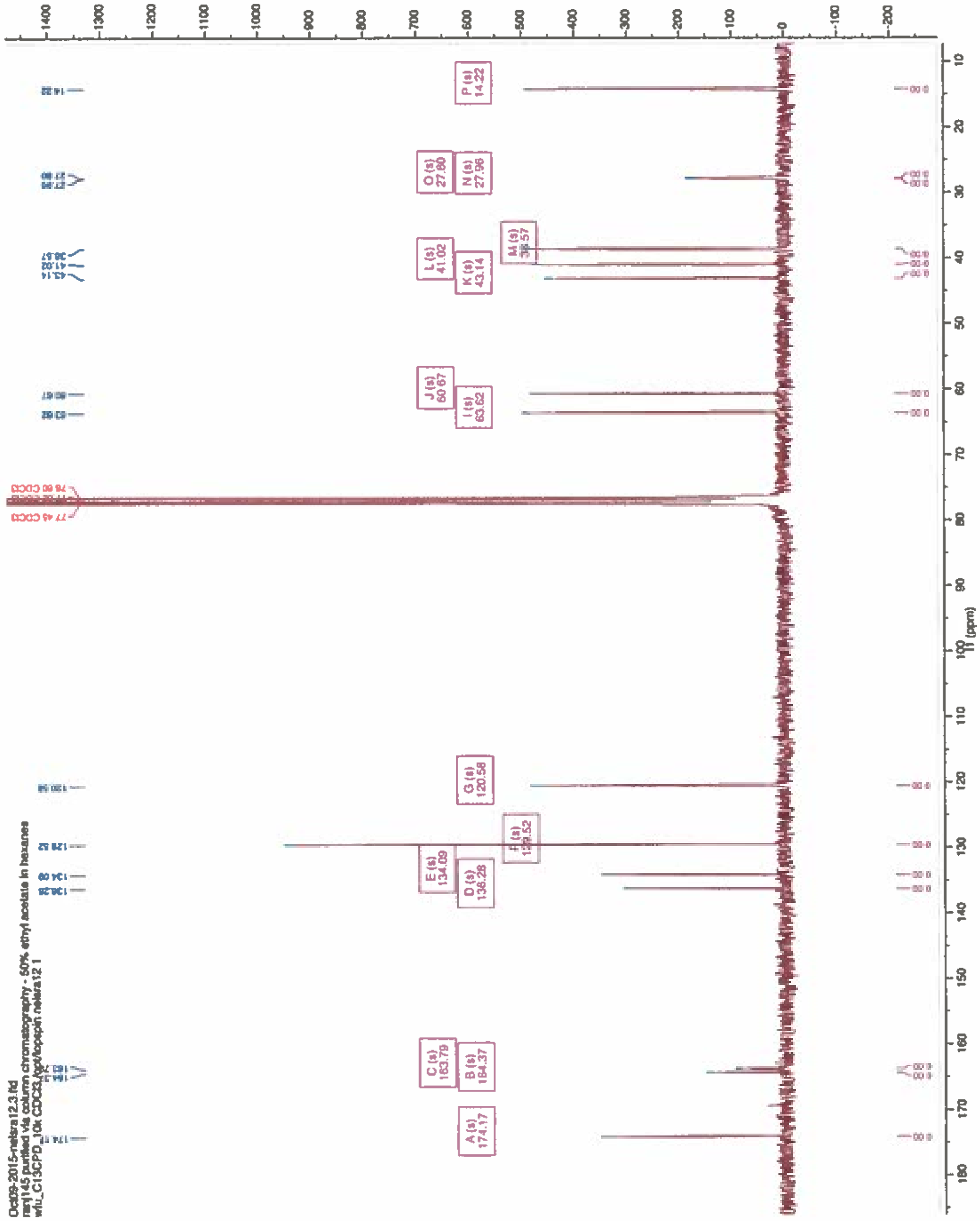

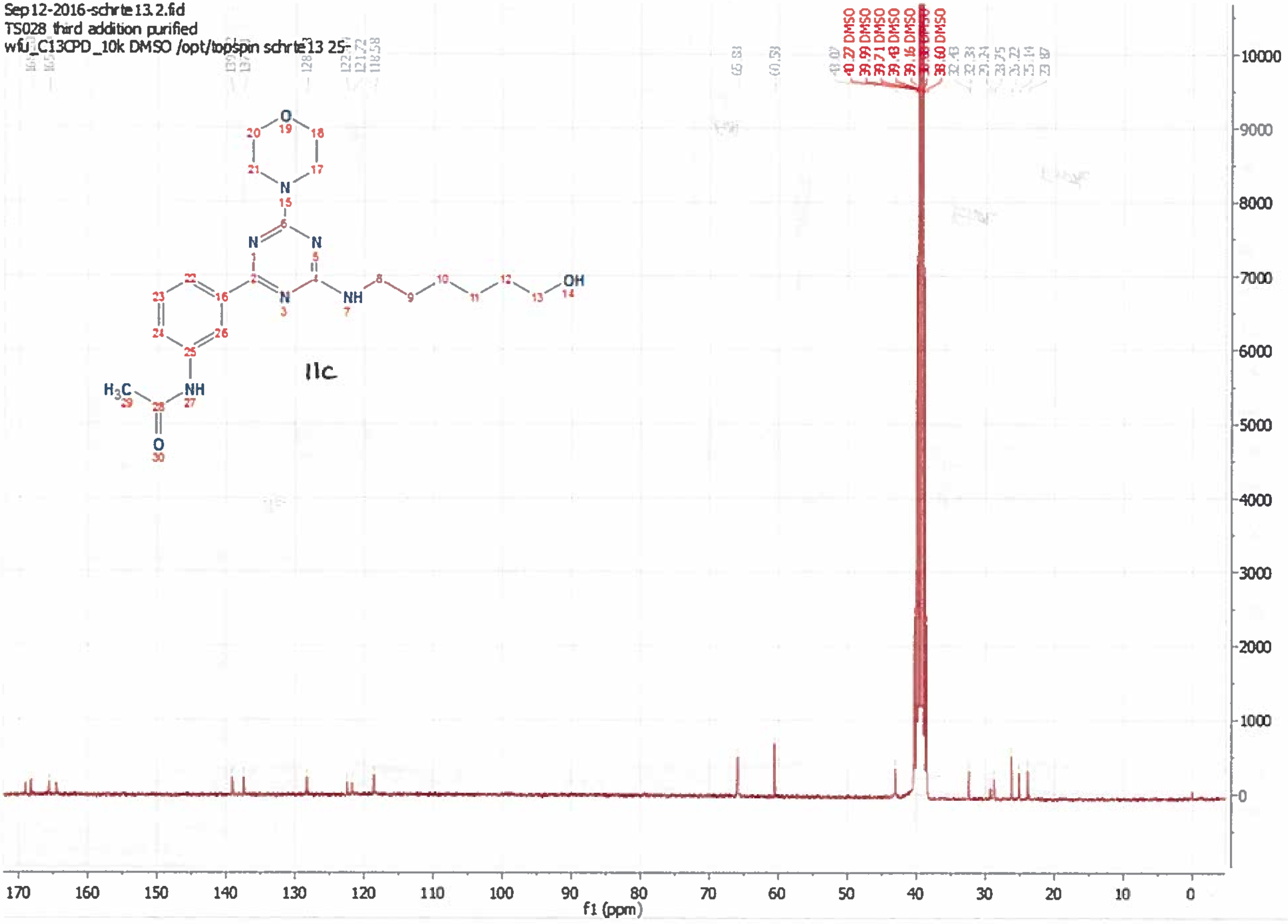
Sep12-2016-schrte13.2.fid
TS028 third addition purified
wfu_C13CPD_10k DMSO /opt/topspin schrte13 25
128.
122.
121.72
118.58
40.27 DMSO
39.99 DMSO
39.71 DMSO
39.43 DMSO
39.16 DMSO
38.60 DMSO
32.43
32.33
11c
f1 (ppm)

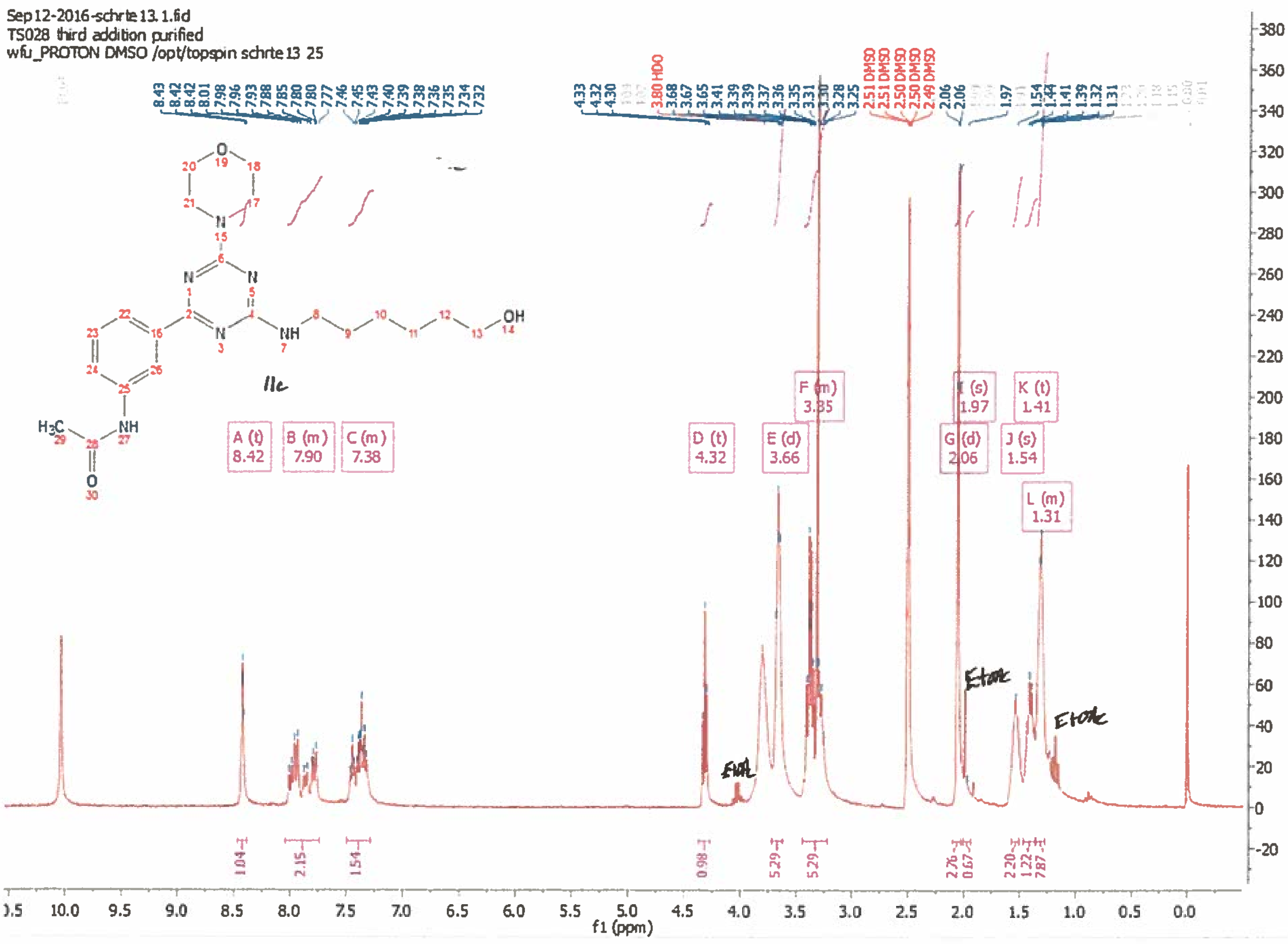
Sep12-2016-schrte13.1.fid
TS028 third addition purified
wfu_PROTON DMSO /opt/topspin schrte13 25
IIc
A (t) 8.42
B (m) 7.90
C (m) 7.38
D (t) 4.32
E (d) 3.66
F (m) 3.35
G (d) 2.06
I (s) 1.97
J (s) 1.54
K (t) 1.41
L (m) 1.31
3.80 HDO
2.51 DMSO
2.51 DMSO
2.50 DMSO
2.50 DMSO
2.49 DMSO
EtOAc
EtOAc
f1 (ppm)

methyl 4-(((4-(2-aminopyrimidin-5-yl)-6-morpholino-1,3,5-triazin-2-yl)amino)methyl)benzoate, 12.

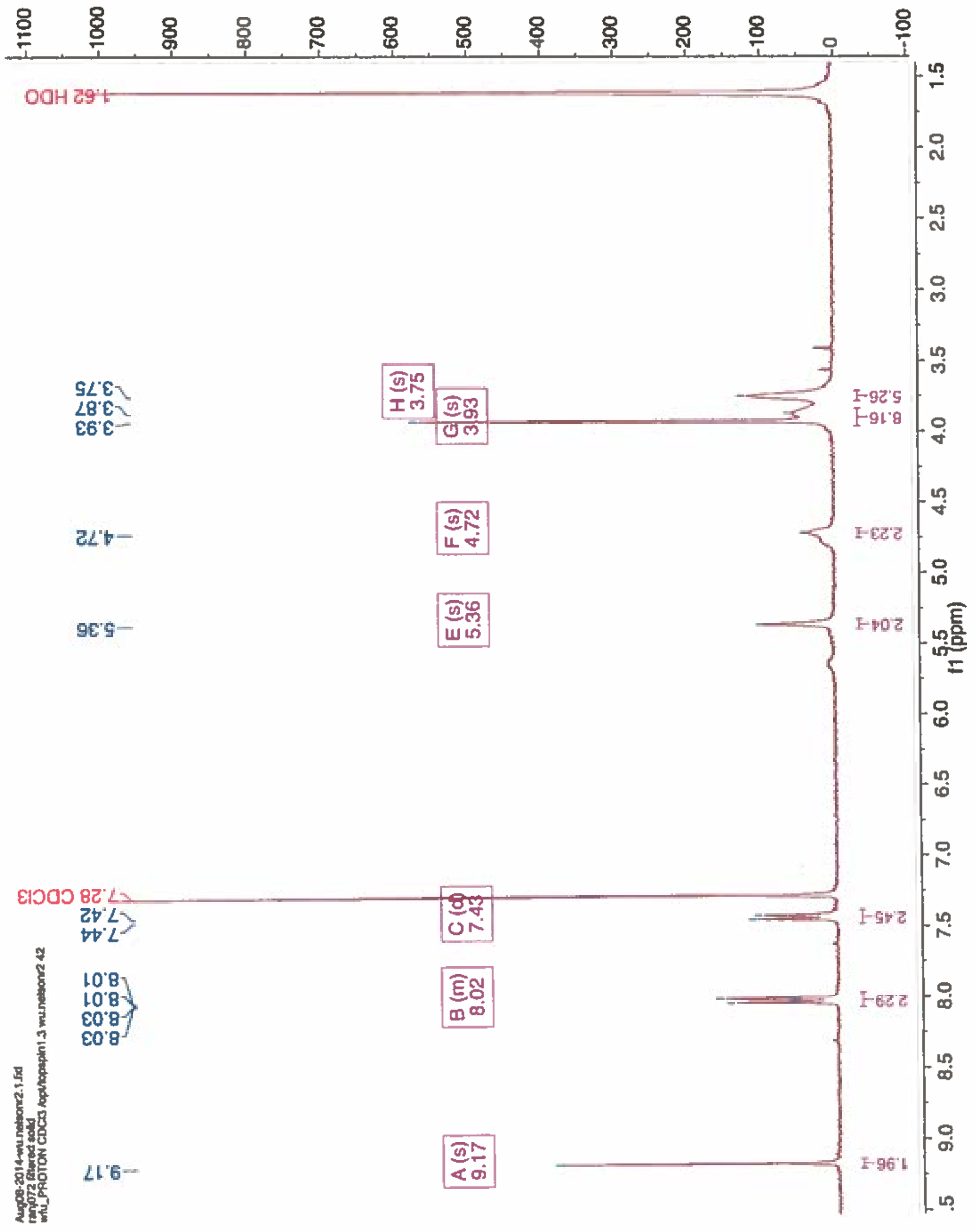

2-(4-((4-(2-aminopyrimidin-5-yl)-6-morpholino-1,3,5-triazin-2-yl)amino)phenyl)ethan-1-ol, 13a.

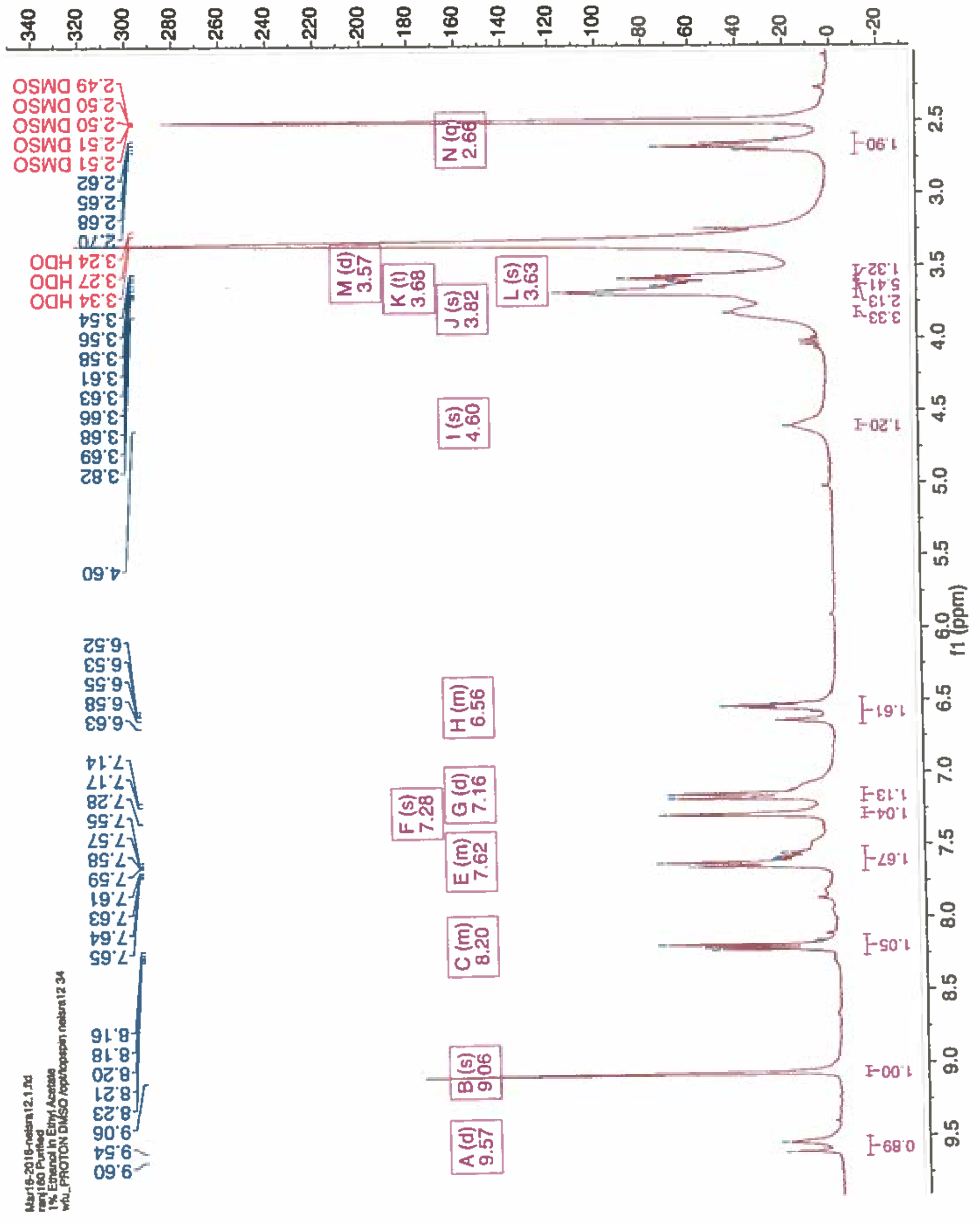

2-(4-((4-(4-aminophenyl)-6-morpholino-1,3,5-triazin-2-yl)amino)phe-nyl)ethan-1-ol, 13b.

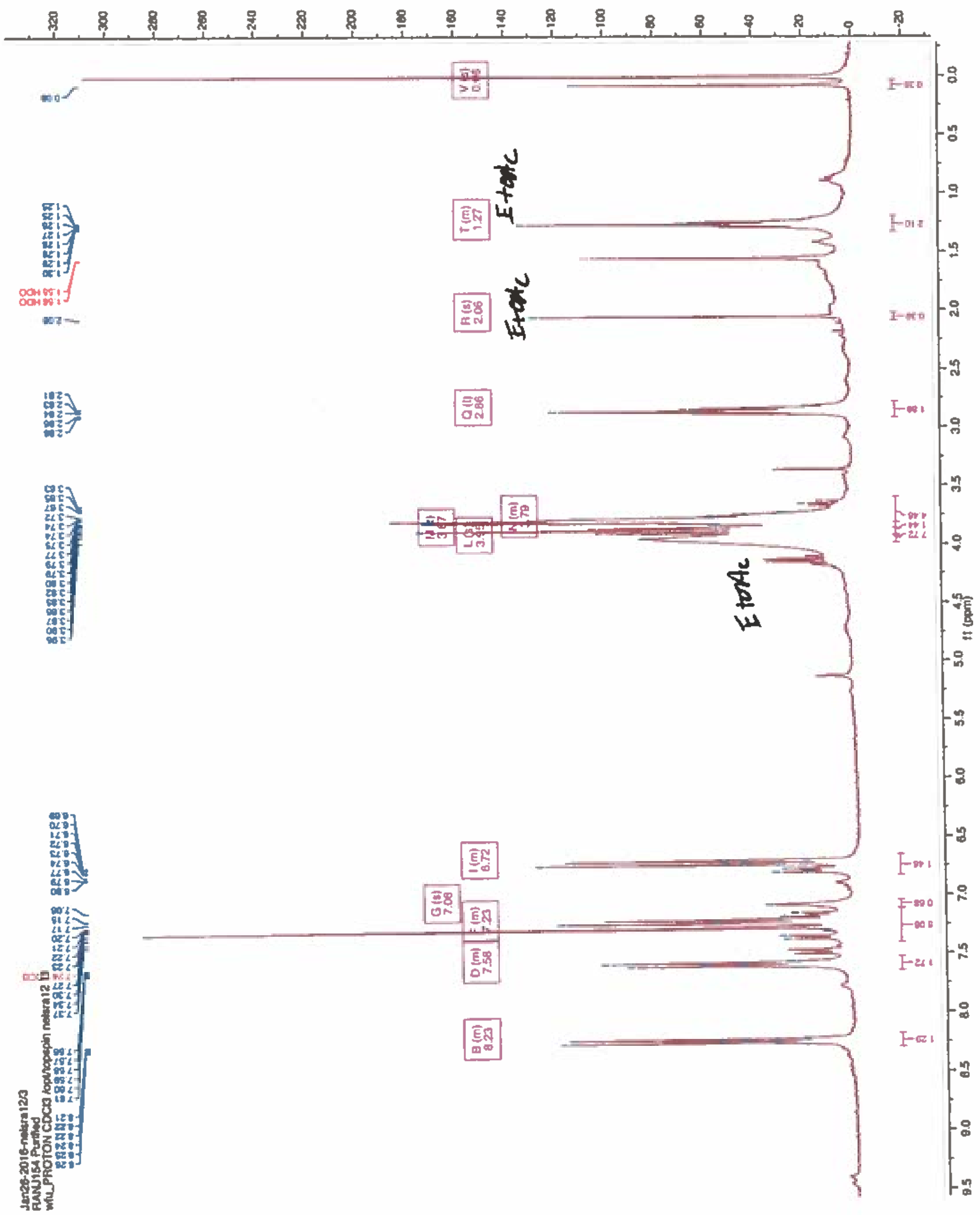

tert-butyl (5-(4-((4-(2-hydroxyethyl)phenyl)amino)-6-morpholino-1,3,5-triazin-2-yl)pyrimidin-2-yl)carbamate, 13c.

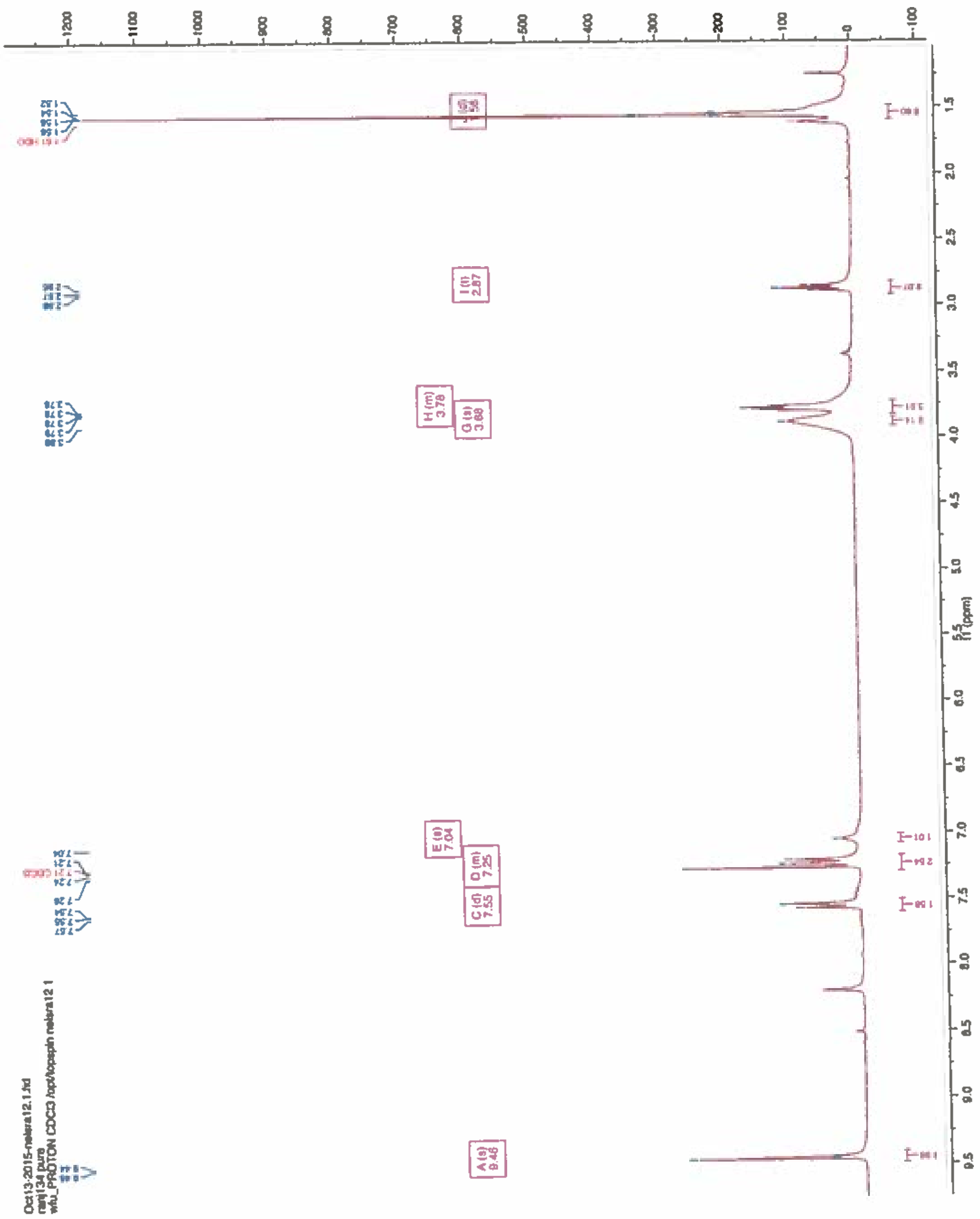

2-(4-((4-(2-methoxypyrimidin-5-yl)-6-morpholino-1,3,5-triazin-2-yl)amino)phenyl)ethan-1-ol, 13d.

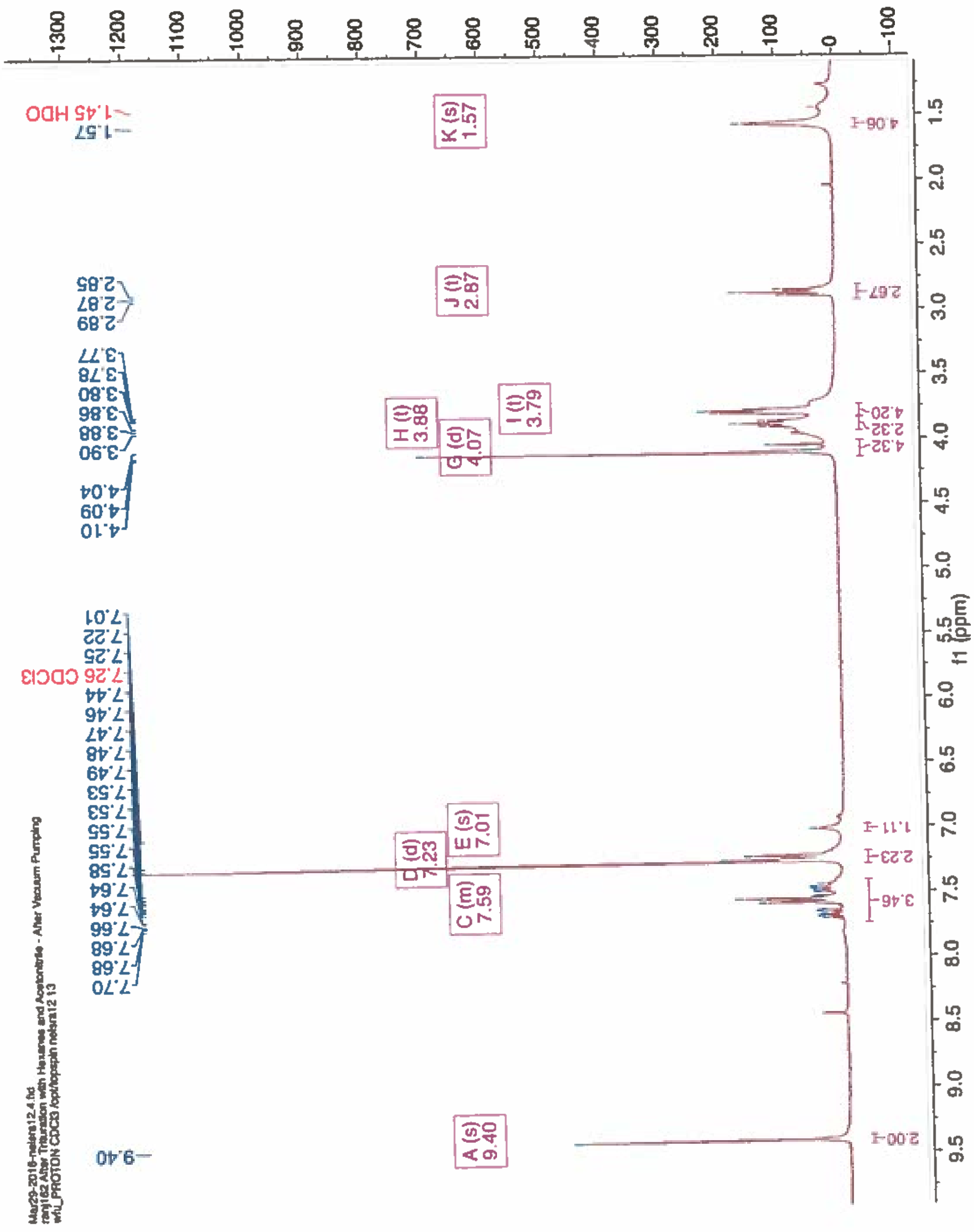

2-(4-((4-morpholino-6-(6-(trifluoromethyl)pyridin-3-yl)-1,3,5-triazin-2-yl)amino)phenyl)ethan-1-ol, 13f.

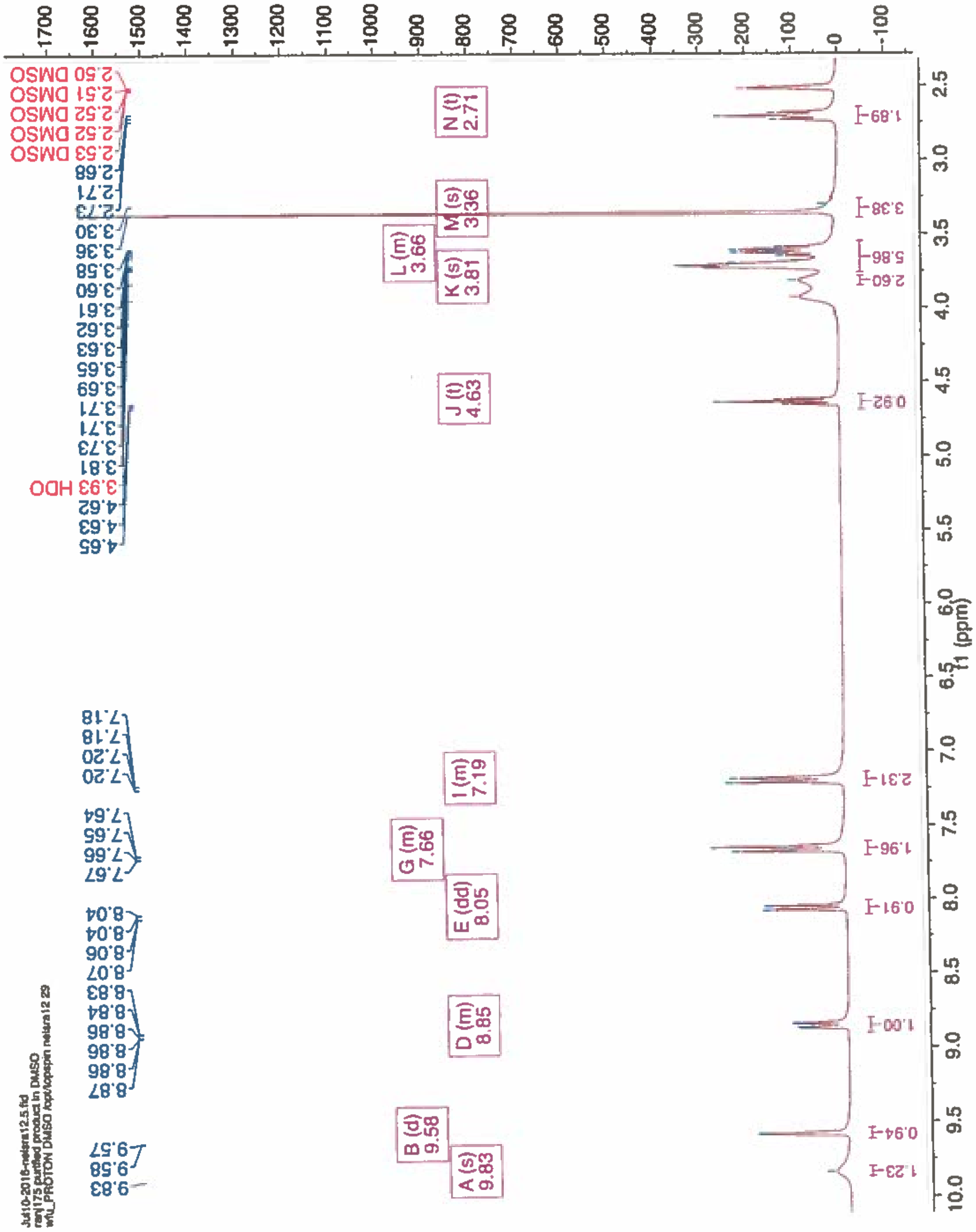

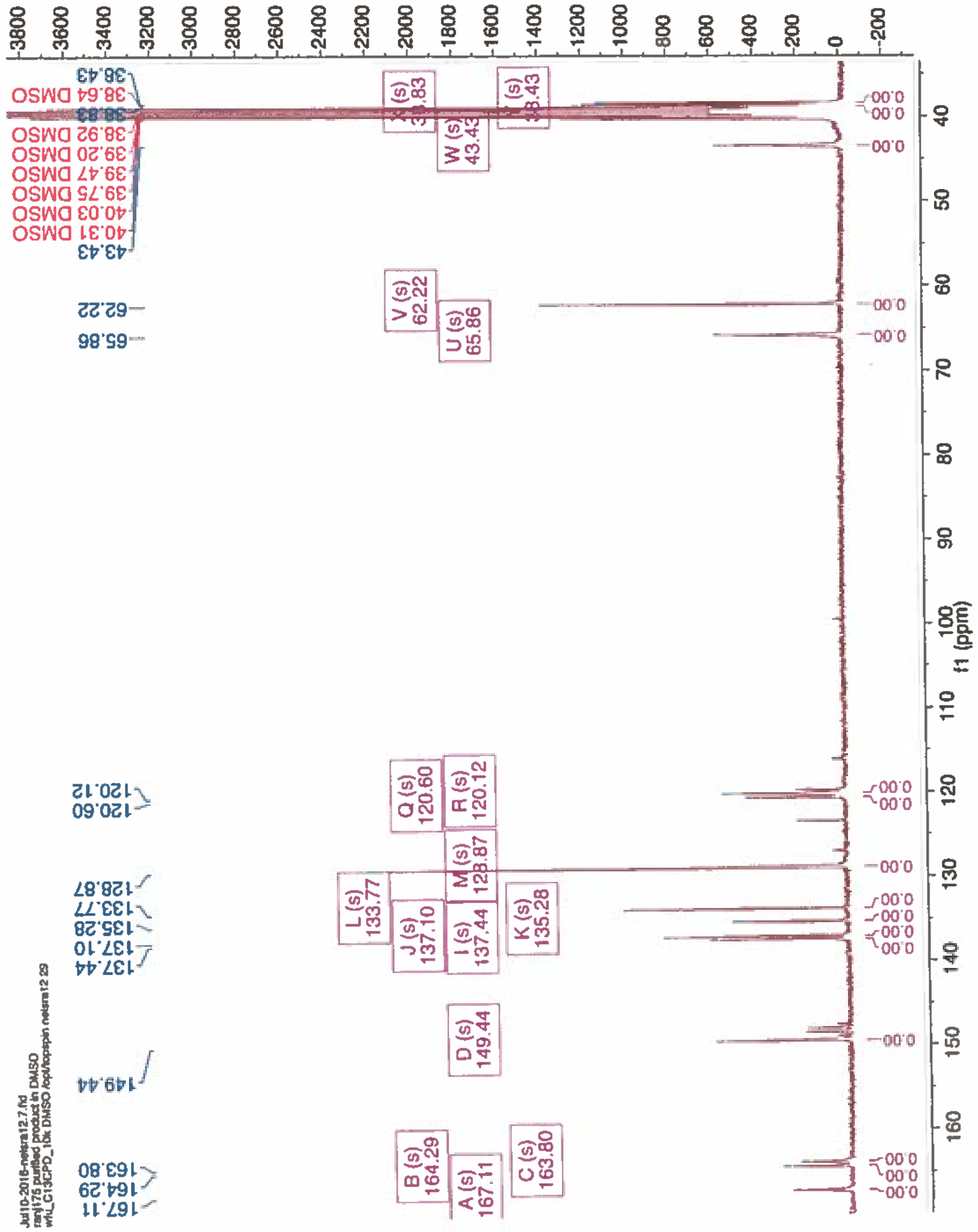
167.11
164.29
163.80
149.44
137.44
137.10
135.28
133.77
128.87
120.60
120.12
65.86
62.22
43.43
40.31 DMSO
40.03 DMSO
39.75 DMSO
39.47 DMSO
39.20 DMSO
38.92 DMSO
38.64 DMSO
38.43
A (s) 167.11
B (s) 164.29
C (s) 163.80
D (s) 149.44
I (s) 137.44
J (s) 137.10
K (s) 135.28
L (s) 133.77
M (s) 128.87
Q (s) 120.60
R (s) 120.12
U (s) 65.86
V (s) 62.22
W (s) 43.43
f1 (ppm)

N-(4-(4-((4-(2-hydroxyethyl)phenyl)amino)-6-morpholino-1,3,5-triazin-2-yl)phenyl)acetamide, 13h.

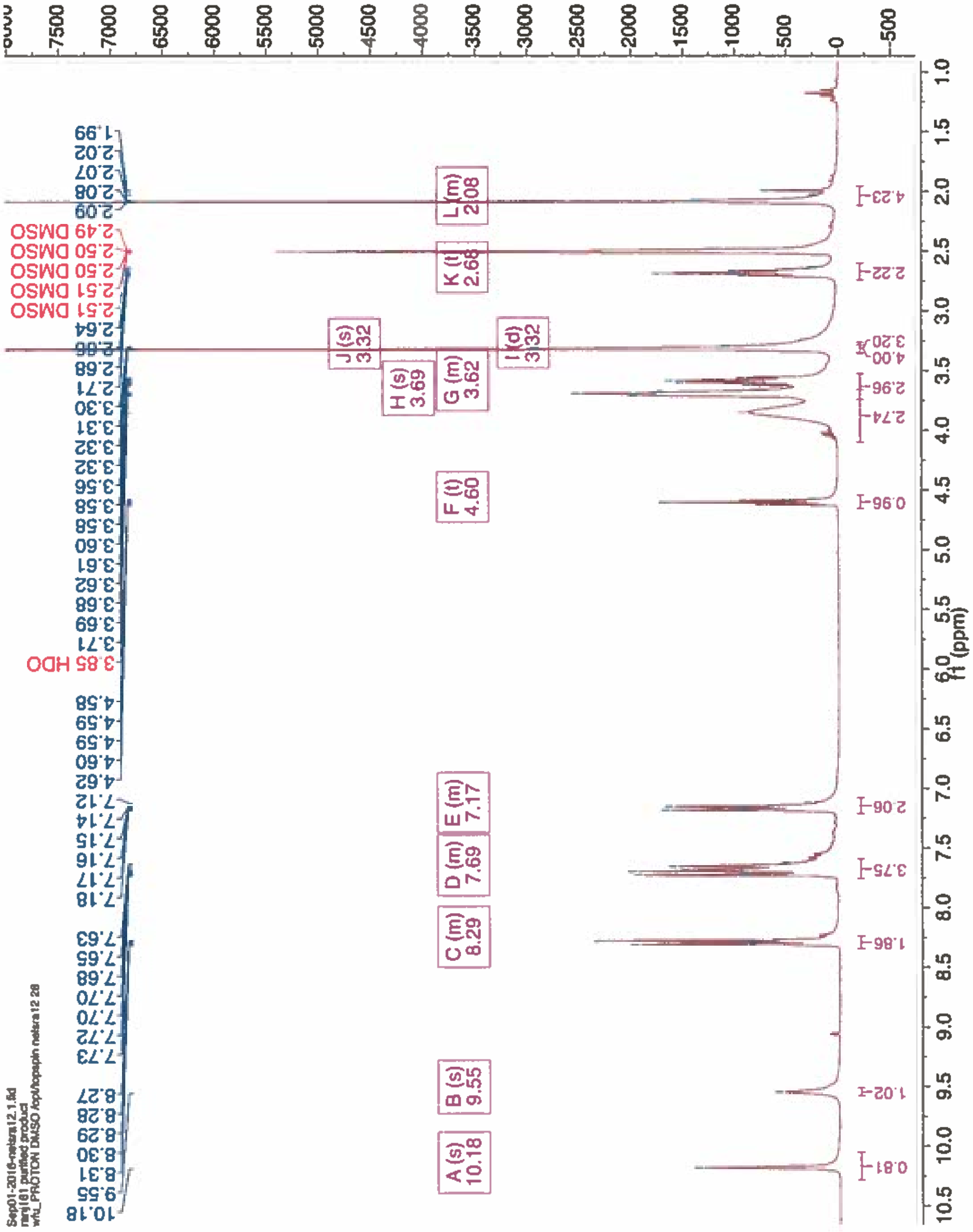

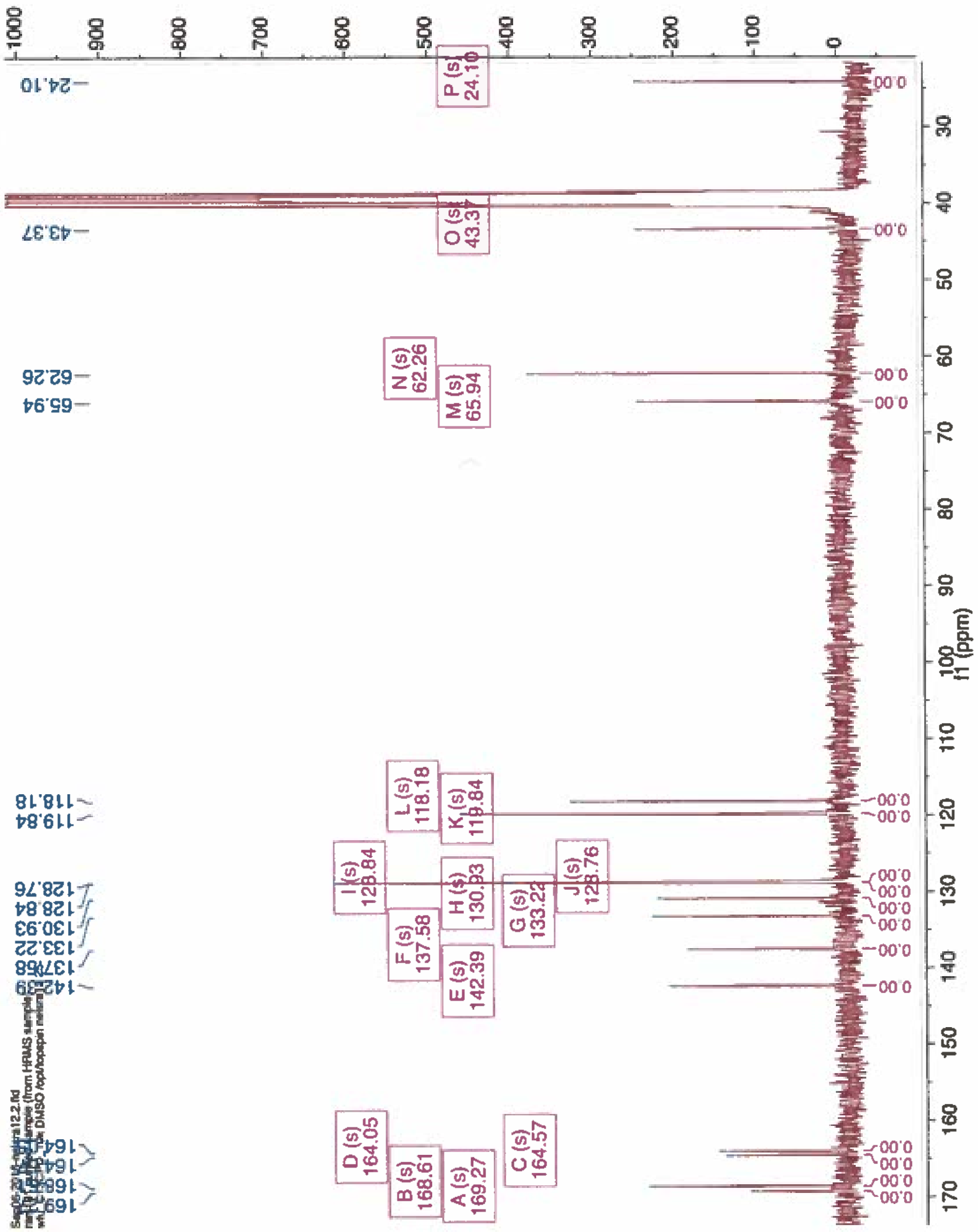

P (s) 24.10
O (s) 43.37
N (s) 62.26
M (s) 65.94
L (s) 118.18
K (s) 119.84
J (s) 128.76
I (s) 128.84
H (s) 130.93
G (s) 133.22
F (s) 137.58
E (s) 142.39
D (s) 164.05
C (s) 164.57
B (s) 168.61
A (s) 169.27
f1 (ppm)

4-((4-chloro-6-morpholino-1,3,5-triazin-2-yl)amino)phenethyl (*tert*-butoxycarbonyl)leucinate, 14.

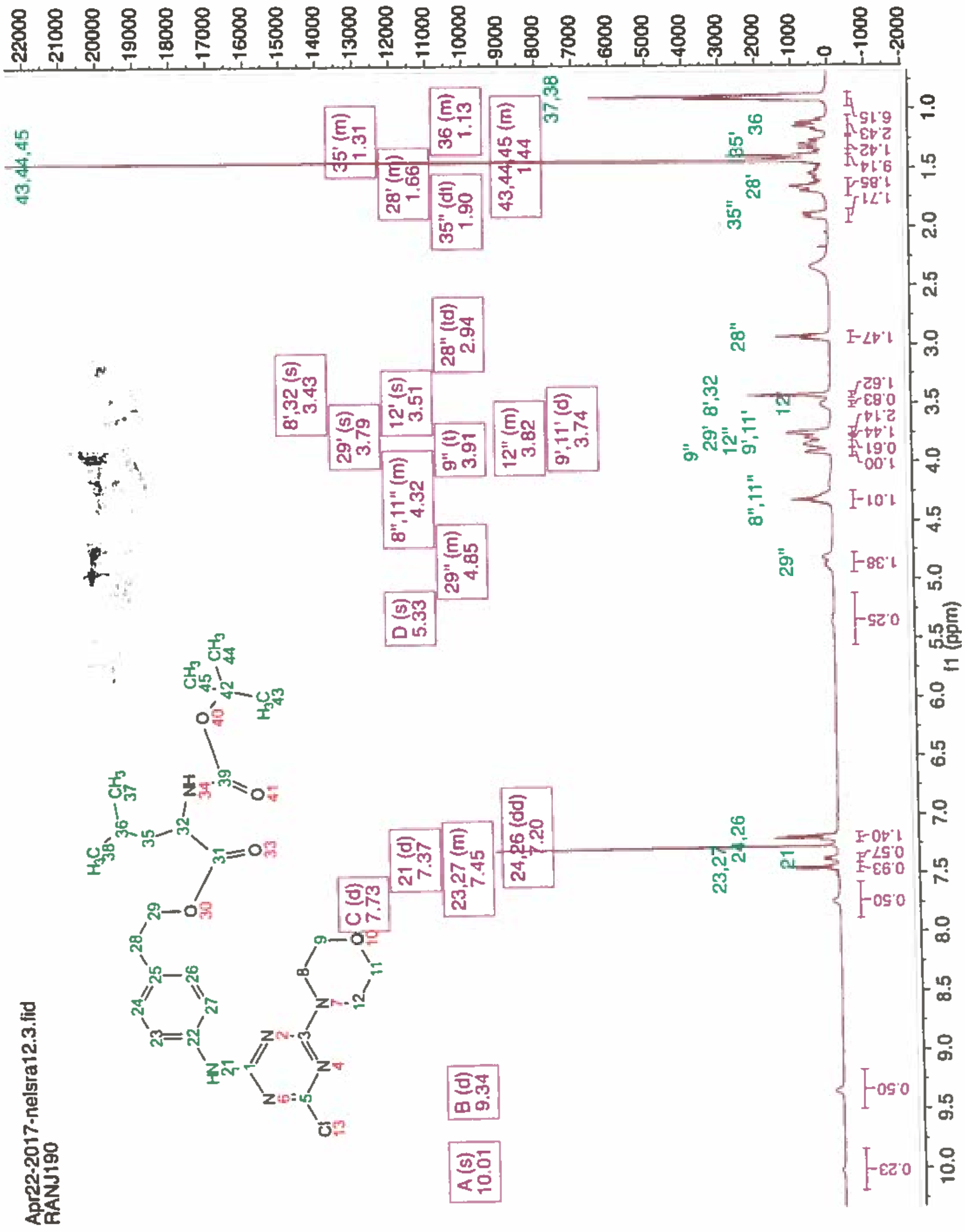

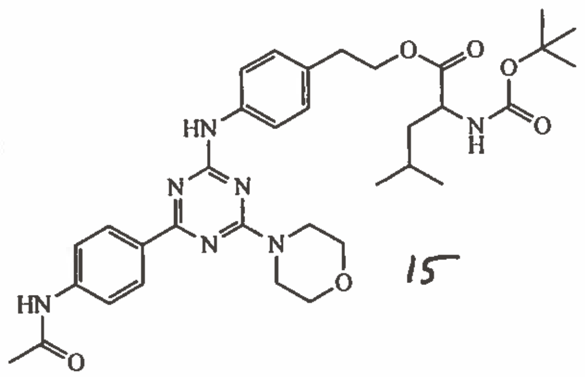

15

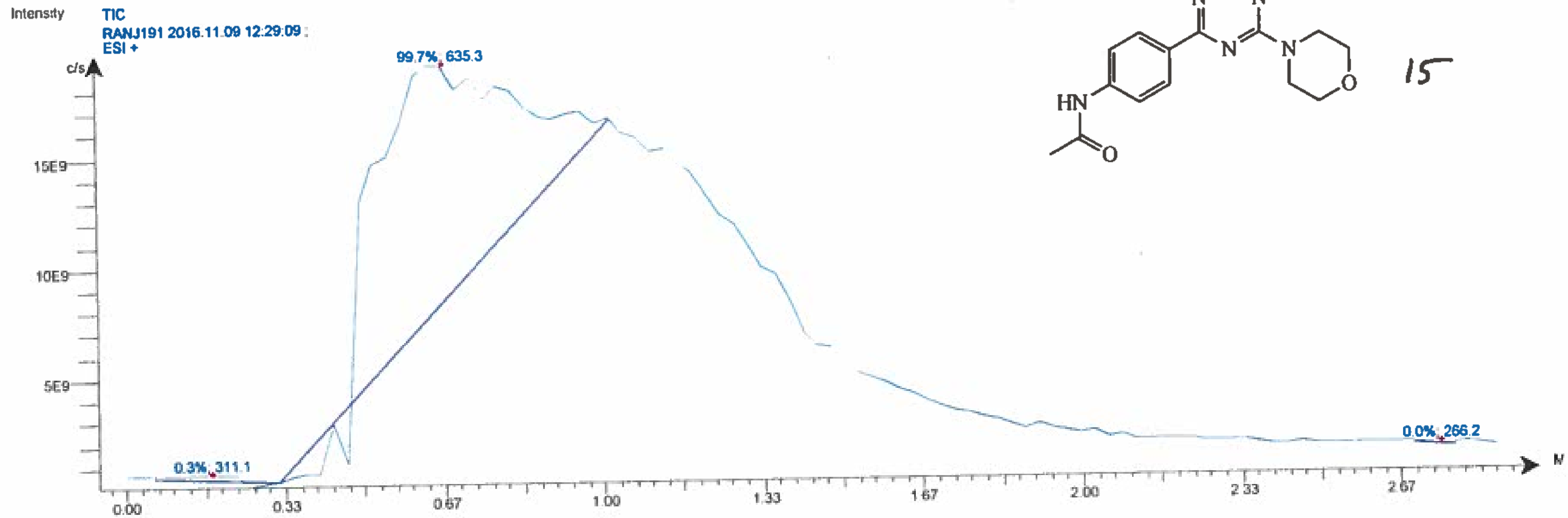

Intensity
TIC
RANJ191 2016.11.09 12:29:09 ;
ESI +
c/s
15E9
10E9
5E9
99.7%, 635.3
0.3%, 311.1
0.0%, 266.2
0.00
0.33
0.67
1.00
1.33
1.67
2.00
2.33
2.67

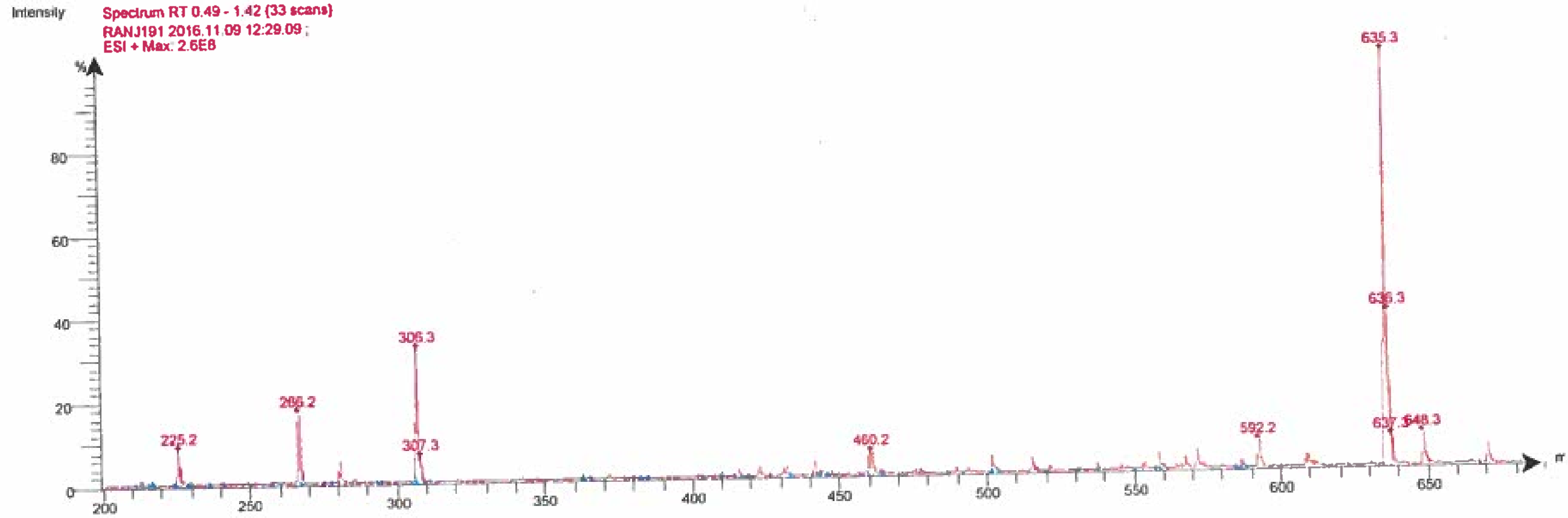

Intensity
Spectrum RT 0.49 - 1.42 (33 scans)
RANJ191 2016.11.09 12:29:09 ;
ESI + Max: 2.6E8
%
80
60
40
20
0
635.3
636.3
306.3
266.2
225.2
307.3
460.2
592.2
648.3
200
250
300
350
400
450
500
550
600
650

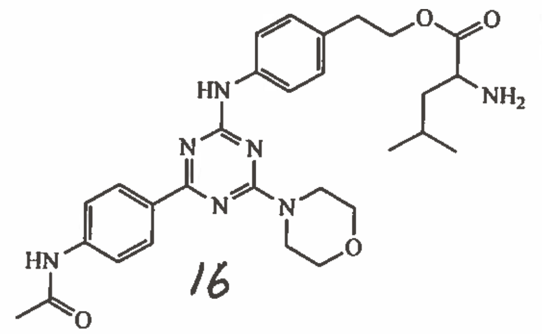
HN
N
N
N
N
O
O
NH2
HN
O
O

16

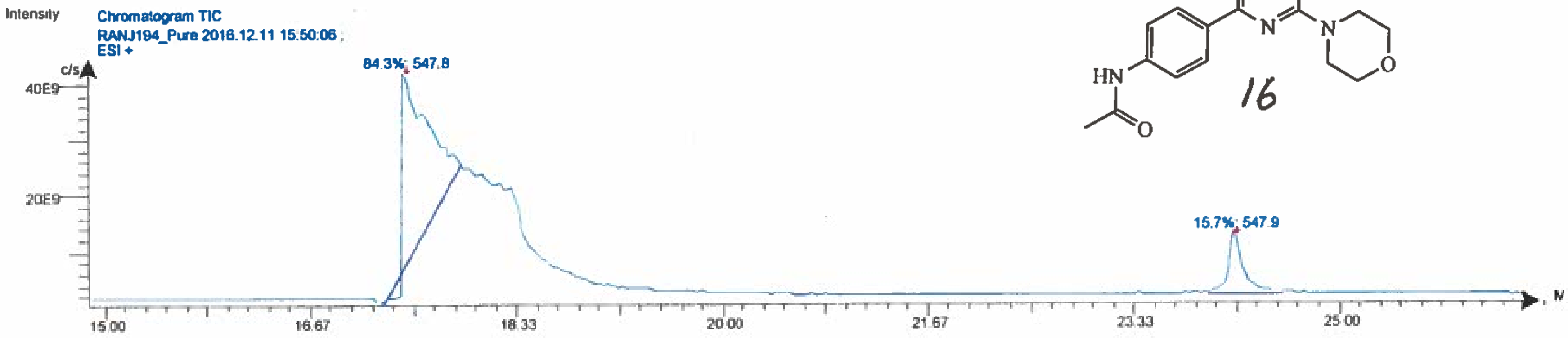
Intensity
Chromatogram TIC
RANJ194_Pure 2016.12.11 15:50:06 ;
ESI +
c/s
40E9
20E9
84.3%; 547.8
15.7%; 547.9
15.00
16.67
18.33
20.00
21.67
23.33
25.00

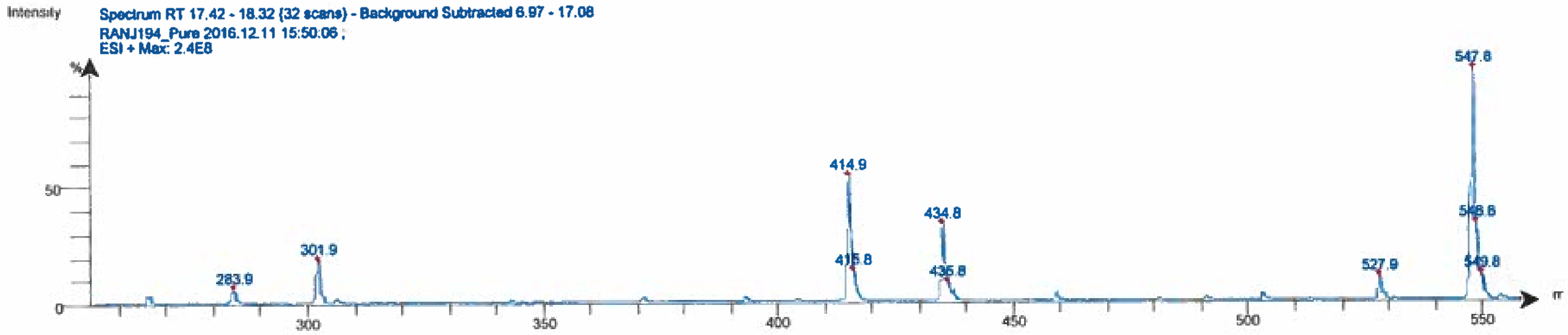
Intensity
Spectrum RT 17.42 - 18.32 (32 scans) - Background Subtracted 6.97 - 17.08
RANJ194_Pure 2016.12.11 15:50:06 ;
ESI + Max: 2.4E8
%
50
0
283.9
301.9
414.9
434.8
527.9
547.8
300
350
400
450
500
550

Qualitative measure of Compound 12's PI3K pathway inhibition by measuring activated p-Akt T308 through a Western blot analysis

Untreated	ZSTK474	Compound **12**			
	10µM	100µM	10µM	1µM	0.1µM

p-Akt T308

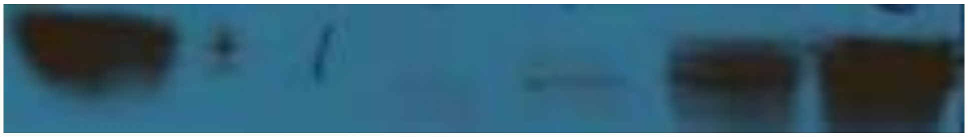

Qualitative measure of Compound 13b's PI3K pathway inhibition by measuring activated p-Akt T308 through a Western blot analysis

Untreated	ZSTK474			Compound **13b**		
	10µM	1µM	0.1µM	10µM	1µM	0.1µM

p-Akt T308

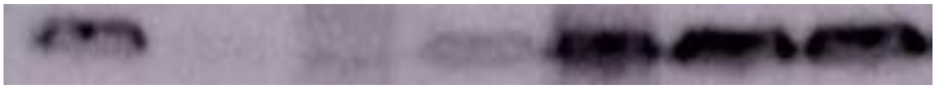

Qualitative measure of Compound 13d's PI3K pathway inhibition by measuring activated p-Akt S473 through a Western blot analysis

Untreated	ZSTK474			Compound **13d**		
	10µM	1µM	0.1µM	10µM	1µM	0.1µM

p-Akt S473

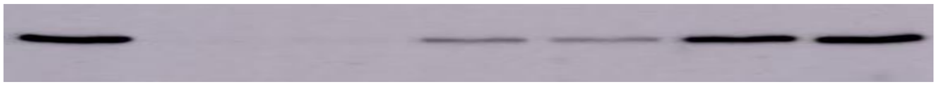

Qualitative measure of Compound 13f's PI3K pathway inhibition by measuring activated p-Akt S473 through a Western blot analysis

Untreated	ZSTK474			Compound **13f**		
	10µM	1µM	0.1µM	10µM	1µM	0.1µM

p-Akt S473

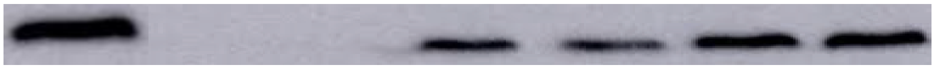

Qualitative measure of Compound 11c's PI3K pathway inhibition by measuring activated p-Akt S476 through a Western blot analysis (RANJ180 = 11c in paper)

10, 1, 0.1 10, 1, 0.1 μM for each

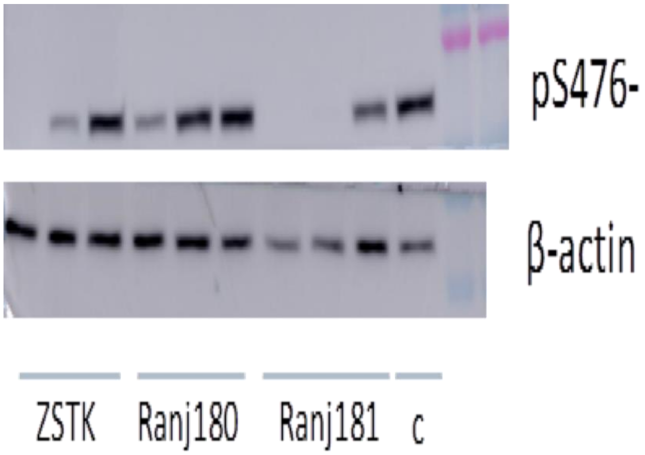

Supplement: Supplementary file 1 [file molecules-23-01628-s001.zip › molecules-323115-SI.pdf]
